# A novel method for sample preparation of fresh lung cancer tissue for proteomics analysis by tumor cell enrichment and removal of blood contaminants

**DOI:** 10.1186/1477-5956-8-9

**Published:** 2010-02-26

**Authors:** Luigi De Petris, Maria Pernemalm, Göran Elmberger, Per Bergman, Lotta Orre, Rolf Lewensohn, Janne Lehtiö

**Affiliations:** 1 Karolinska Biomics Center, Department of Oncology and Pathology, Karolinska Institutet, Stockholm, Sweden; 2 Department of Pathology, Karolinska University Hospital Solna, Stockholm, Sweden; 3 Department of Cardiothoracic Surgery and Anesthesiology, Karolinska University Hospital Solna, Stockholm, Sweden

## Abstract

**Background:**

In-depth proteomics analyses of tumors are frequently biased by the presence of blood components and stromal contamination, which leads to large experimental variation and decreases the proteome coverage. We have established a reproducible method to prepare freshly collected lung tumors for proteomics analysis, aiming at tumor cell enrichment and reduction of plasma protein contamination. We obtained enriched tumor-cell suspensions (ETS) from six lung cancer cases (two adenocarcinomas, two squamous-cell carcinomas, two large-cell carcinomas) and from two normal lung samples. The cell content of resulting ETS was evaluated with immunocytological stainings and compared with the histologic pattern of the original specimens. By means of a quantitative mass spectrometry-based method we evaluated the reproducibility of the sample preparation protocol and we assessed the proteome coverage by comparing lysates from ETS samples with the direct lysate of corresponding fresh-frozen samples.

**Results:**

Cytological analyses on cytospin specimens showed that the percentage of tumoral cells in the ETS samples ranged from 20% to 70%. In the normal lung samples the percentage of epithelial cells was less then 10%. The reproducibility of the sample preparation protocol was very good, with coefficient of variation at the peptide level and at the protein level of 13% and 7%, respectively. Proteomics analysis led to the identification of a significantly higher number of proteins in the ETS samples than in the FF samples (244 vs 109, respectively). Albumin and hemoglobin were among the top 5 most abundant proteins identified in the FF samples, showing a high contamination with blood and plasma proteins, whereas ubiquitin and the mitochondrial ATP synthase 5A1 where among the top 5 most abundant proteins in the ETS samples.

**Conclusion:**

The method is feasible and reproducible. We could obtain a fair enrichment of cells but the major benefit of the method was an effective removal of contaminants from red blood cells and plasma proteins resulting in larger proteome coverage compared to the direct lysis of frozen samples. This sample preparation method may be successfully implemented for the discovery of lung cancer biomarkers on tissue samples using mass spectrometry-based proteomics.

## Background

Lung cancer is the number one cause of cancer-related deaths worldwide [1]. According to clinico-pathological criteria lung cancer can be divided into two major groups: small-cell lung cancer (SCLC) and non-small-cell lung cancer (NSCLC). The latter accounts for 85% of all lung cancer cases, and further comprises diverse histological subtypes, such as adenocarcinoma, squamous-cell carcinoma and undifferentiated large-cell carcinoma. All NSCLC subtypes share some common features, including a marked resistance to anticancer drugs, lack of effective screening strategies to diagnose the disease when it still is at a potentially curable early stage, and an unacceptably low overall survival, with as few as 15% of patients still alive after 5 years from the diagnosis.

Considering this survival rate, it is clear that there is a strong need to more deeply understand the biology of lung cancer, to develop novel therapeutics and to identify reliable biomarkers that can either be used for early diagnosis, to accurately predict the response of lung tumors to therapy or to foresee the natural history of the disease in terms of metastatic potential and invasiveness.

Proteomics technologies are currently being developed and applied on lung cancer tissue specimens to perform discovery-based research. Most studies that either implemented gel-based or mass spectrometry (MS)-based proteomics have extracted proteins by direct lysis of archival fresh-frozen tumor tissues [2-6]. However, lung cancer tissue is heterogeneous, containing not only tumor cells, but also various degrees of necrotic areas, inflammatory infiltrate, stromal and vascular components and can in addition be highly contaminated by red blood cells and plasma proteins. Overall, these components can have a deep confounding effect on proteomics experiments, limiting the resolution of the analytical methods in discovering tumor specific biomarkers.

In this study we aimed at developing a reproducible sample preparation method that would generate cell suspensions free from contamination of plasma and erythrocyte proteins as well as stromal components. To evaluate this, we performed cytological and proteomics analyses on samples of lung tumors and normal lung parenchyma and compared our method with a direct lysis of corresponding fresh frozen samples taken from the same surgical specimens.

## Methods

### Collection of tissue specimens

Samples were collected at the Unit of Thoracic surgery, department of Cardiothoracic Surgery and Anesthesiology, Karolinska University Hospital, Stockholm, Sweden.

Cases included in the present study were two adenocarcinomas, two large-cell carcinomas, two squamous-cell carcinomas and two samples of normal lung parenchyma.

To determine the reproducibility of the sample preparation method, an additional case of lung adenocarcinoma was included.

The study was approved by the Ethics board at Karolinska University Hospital, Stockholm, and written informed consent was obtained from all patients.

### Sample preparation and protein extraction

Intact surgical specimens were placed in ice immediately after surgical resection. Within 30 min the specimens were macroscopically examined by a pathologist who took a biopsy from a region representative of the tumor. The biopsy was cut in two, one piece was snap frozen in liquid nitrogen and the other half was placed in phosphate buffer saline solution (PBS) containing a protease inhibitor cocktail (Roche Diagnostic, Mannheim, Germany). A control tumor biopsy was taken from the juxtaposed tumor tissue, fixed in formalin and embedded in paraffin. Hematoxylin-eosin (H&E) staining of this sample was used to evaluate the morphology and cell content of the specimens later prepared for proteomics analyses. A similar procedure was conducted for collecting samples of histologically normal lung parenchyma.

To obtain enriched tumor cell suspensions (ETS), approximately 100-300 mg of fresh tissue samples were homogenized with a motor-driven tissue homogenizer (Ika-Werke, Staufen, Germany) followed by filtration through a 70 μm cell strainer (BD Biosciences, Erembodegem, Belgium). The entire preparation protocol was conducted on ice. The filtrated homogenates were centrifuged at 200 × g for 10 min. After removal of the supernatant, pellets were dissolved in 5 ml hemolysis buffer (154 mM NH_4_Cl, 10 mM KHCO_3_, 0.1 mM EDTA, pH 7.2), incubated for 8 min, washed at 200 × g for 10 min, again resuspended in 1 ml hemolysis buffer and incubated for 5 min. After a final washing at 200 × g for 10 min pellets were solubilized in PBS. An aliquot was diluted till the final volume of 1 ml and transferred to 8 cytospin cups (100 μl of suspension per cup). These were centrifuged at 700 rpm for 3 min at +4°C.

Microscope slides with cytospin specimens were air dried and frozen at -20°C. The remaining sample was centrifuged at 300 × g for 10 min, the supernatant was discarded and the pellet frozen at -80°C.

To determine the reproducibility of the sample preparation protocol, one specimen of lung adenocarcinoma was prepared as described above. However, after the mechanical mincing and filtration steps, the flowthrough was divided into 5 aliquots that were then processed in parallel, generating five separate replicates.

Proteins from the ETS samples were extracted by solubilizing the cell pellets obtained as described above in approximately ten sample volumes of lysis buffer (urea 8 M and Chaps 1% in PBS) for 30 min on ice. The samples were then centrifuged at 10000 × g for 10 min at +4°C and supernatants were used for proteomics analyses.

Frozen samples (approximately 20 mg) were cut in small pieces, transferred to Eppendorf tubes containing approximately ten sample volumes of lysis buffer (see above) and incubated for 30 min in ice trying to grossly disrupt the tissue specimens with the help of a plastic mortar. Samples were thereafter centrifuged at 10000 × g for 10 min at +4°C and supernatants were used for proteomics analyses.

### Immunocytochemistry (ICC)

Cytospin specimens on slides were fixed with cold acetone for 10 min. Slides were incubated with primary antibodies (ABs) for 30 min at room temperature in humidity chamber, followed by two further incubations with a secondary AB rabbit anti-mouse (30 min) and a tertiary AB swine anti-rabbit (30 min) both conjugated with alkaline phosphatase. Tris Buffered Saline (TBS) solution pH 7.6 was used for washing steps as appropriate. After developing and nuclear counterstaining with hematoxylin, slides were dried in air and mounted.

The following primary mouse monoclonal antibodies were used: cytokeratin clone MNF 116, CD45 (leukocyte common antigen), CD68 (clone PG-M1), Smooth Muscle Actin (SMA clone 1A4).

All antibodies, including the secondary and tertiary antibodies, were pursued from DakoCytomation (Glostrup, Denmark)

To evaluate cell morphology, cytospin specimens were stained with Giemsa.

### Evaluation of histological and cytological specimens

Histological sections were examined to determine the cell morphology and the structure of the primary lung tumors included in the study, and to determine the degree of necrosis and inflammatory infiltration. The percentage of necrosis in the tumors was scored as; absent, <10%, 10-30% and >30%. Infiltration of inflammatory cells was graded on a 1-3 scale where 1 represented minimal, 2 represented moderate and 3 represented intense inflammatory infiltration.

In terms of the quality and relative quantity of diverse cell types that may compose the final pellets from the ETS preparation, cytospin specimens were evaluated. Cell morphology was assessed with Giemsa staining. In addition, ICC was used to quantify the percentage of diverse cell populations in terms of epithelial cells (cytokeratin MNF 116+), leukocytes (CD45+), macrophages (CD68+) and stromal components (SMA+).

Histology and cytology specimens were evaluated by a specialist in lung cancer pathology.

### Sample preparation for proteomics analyses

One hundred microgram of protein from the fresh-frozen (FF) and enriched tumor-cell suspension (ETS) samples and eighty microgram of the reproducibility samples were precipitated using ice-cold acetone (Chromasolv^®^, Sigma-Aldrich, Steinheim, Germany). Four sample volumes of ice-cold acetone were added and the samples were then incubated in -20°C for 30 minutes. The samples were then centrifuged at +4°C for 10 minutes, the supernatant was then disregarded and the pellet was allowed to air-dry.

Precipitated samples were dissolved in iTRAQ dissolution buffer and digested according to manufacturer's instructions (Applied Biosystems, Forster City, CA, USA). Samples were then pooled and labelled with eight-plex iTRAQ reagent. The labelling strategy is shown in table 1.

**Table 1:** Labeling strategy for the iTRAQ samples.

Pooled sample	iTRAQ reporter ion							
	113	114	115	116	117	118	119	121
**ETS reproducibility**	Repl 1	Repl 2	Repl 3	Repl 4	Repl 5	Repl 5	Repl 5	Repl 5
**ETS preparation**	Large-cell 1	Large-cell 2	Adk 1	Adk 2	SCC 1	SCC 2	Normal 1	Normal 2
**FF preparation**	Large-cell 1	Large-cell 2	Adk 1	Adk 2	SCC 1	SCC 2	Normal 1	Normal 2

(Abbreviations: Repl, replicate; Adk, adenocarcinoma; SCC, squamous-cell carcinoma).

Digests were applied to 1 ml Strata X-C 33 μm polymeric strong cation exchange (SCX) microcolumns (Phenomenex, Torrance, CA, USA). The microcolumns were initially washed with 1 ml 100% methanol (Chromasolv^®^, Riedel-de-Haën, Seelze, Germany) followed by 1 ml MilliQ grade water. The sample was adjusted to 500 μl 0.1% formic acid (puriss, Sigma-Aldrich) and then applied to the columns. After a wash with 1 ml 30% methanol and 0.1% formic acid the samples were eluted with 30% methanol and 5% ammonium hydroxide (33% solution puriss, Riedel-de-Haën). Samples were then dried in a SpeedVac system.

For iso-electric focusing (IEF), approximately 600 μg of ETS and FF pooled iTRAQ labelled samples were dissolved in 225 mL 8 M urea. Dry sample application gels were kindly supplied by GE Healthcare Bio-Sciences AB, Uppsala, Sweden. The application gels were rehydrated in sample overnight while the strips (pH 3.7-4.9) were rehydrated overnight in 8 M urea and 1% Pharmalyte™ 2.5-5 (GE Healthcare Bio-Sciences AB). The IPG strips were put in the focusing tray and the application gels containing the samples were placed on the anodic end of the IPG strips with filter paper between the application gels and the electrodes. The compatibility of iTRAQ labelling with IEF by IPG has been previously shown by our group [7]. The samples were then focused as described in [8]. After focusing the strips were cut in 24 pieces starting at the acidic end, as described in [9]. Peptides were then eluted in two steps. First, 240 μl 0.1% trifluoro acetic acid (TFA) (puriss, Sigma-Aldrich) were added to each piece from the strip and incubated for 2 h on a shaking board. Two-hundred and forty μl 0.1% TFA 100% acetonitrile (Grade S, Rathburn Chemicals Ltd, Walkerburn, Scotland) were then added to each of the tubes. After 3 h incubation on shaking board the passive elution solution was collected. Samples were then freeze dried in SpeedVac and kept at -20°C until analysis.

### LC/MS/MS analysis

Freeze dried IPG fractions were dissolved in 16 μl 0.05% heptafluorobutyric acid (HFBA) (puriss, Fluka, Steinheim Germany) and applied to an Ultimate 3000 HPLC system (LC-Packings, Sunnyvale, CA, USA) using μl-pickup. 0.05% HFBA was used as loading solvent as well as transport liquid.

Monolithic trap cartridge, 200 μm × 5 mm PS-DVB (LC-Packings), was used for desalting and concentration and followed by an analytical monolithic column, PS-DVB 200 μm (LC-Packings). The flow rate was set to 1.5 μl/min. Solvent A was 3% acetonitrile 0.05% TFA (v/v) and solvent B was 80% acetonitrile 0.04% TFA (v/v). Peptides were separated using the following gradient: 0-8 min 0% (v/v) B, 8-9 min 0-10% (v/v) B, 9-40 min 10-55% (v/v) B, 40-45 min 55-95% (v/v) B, 45-55 min 0% (v/v) B. The Probot fraction collector was set to collect fractions every 6 s between 10-40 min onto a blank matrix-assisted laser desorption ionization (MALDI) target plate (Applied Biosystems, Forster City, CA, USA). The eluent was mixed 1:1 (v/v) post column with 7 mg/ml α-cyano-4-hydroxycinnamic acid (CHCA) (Bio-Rad Laboratories, Hercules, CA, USA) in 70% acetonitrile before spotted onto the MALDI target. A 4800 MALDI TOF-TOF (Applied Biosystems) instrument was used to analyze the samples. Before analysis the plate was externally calibrated in MS and MS/MS mode. Using the 4000 Series Explorer TM Software v.3.5.28193 (Applied Biosystems) a maximum of 15 precursors with s/n over 100 was set to be picked from each spot and 1000 shots in the range 700-4000 *m/z *were collected for each MS spectrum. MS/MS was performed averaging 3000 shots.

All MS/MS data obtained were submitted to ProteinPilot (Applied Biosystems) for database searching and iTRAQ reporter ion quantification. The searches were performed against a target/decoy database based on the IPI human 3.54 (20081125) protein sequence database, using a 95% confidence cut off limit. Biological modifications as well as amino acid substitutions were allowed for in the search. False discovery rate (FDR) and precursor mass accuracy was calculated based on all peptides ≥ 95% confidence.

### Data analysis

To compare the two sample preparation approaches, all proteins identified with ≥ 95% confidence were analyzed with ProteinCenter (Software Version 2.6.0, Build RELEASE_2_6_0.1 Data Version 110, Proxeon Bioinformatics A/S, Odense, Denmark).

Reproducibility of the single cell suspension preparation was calculated based on iTRAQ intensities, both on a peptide level and on a protein level, using all peptides and proteins ≥ 95% confidence. Reproducibility was calculated on two levels, both for the digestion and MS analysis workflow alone and for the entire cell suspension preparation workflow. Protein concentration was estimated using protein sequence coverage as calculated in ProteinPilot (Applied Biosystems). Only proteins containing the iTRAQ label were used in the protein abundance analysis.

## Results

### Microscopic analysis of cell content

The evaluation of histological and cytological cases is summarized in Table 2.

**Table 2:** Summary of cytological and histological evaluation of cases included in the study

Case	Large-cell 1	Large-cell 2	Adk 1	Adk 2	SCC 1	SCC 2	Normal 1	Normal 2
Histology								
Necrosis	10-30%	10-30%	Absent	<10%	>30%	<10%		
Inflammatory infiltration	Intense	Intense	Minimal	Intense	Minimal	Intense		

Cytology on cytospin specimens								
Giemsa % tumor cells or epithelial cells	<50%	40%	90%	30%	n.e.	90%	Few	Few
MNF116	50%	40-50%	20%*	30%*	n.e.	60-70%	5-10%	10%
CD45	50%	50%	10%	30%	n.e.	40%	90%	90%
CD68	10%	20%	5%	10%	n.e.	10%	70%	40%
SMA	<5%	<5%	20%	5%	n.e.	10%	5%	<5%

Abbreviations: Adk adenocarcinoma, SCC squamous cell carcinoma; n.e. non-evaluable

* In adenocarcinoma samples, approximately 30% of "nude" nuclei were observed (see text)

Cytospin specimens were not evaluable in one of the two squamous-cell carcinoma cases. This could be due to an excessive dilution of the ETS aliquot resulting in the deposition of scarce material on the microscope glasses.

The evaluation of normal lung tissue showed that the ETS samples contained mainly inflammatory cells and very few lung and bronchial epithelial cells.

In the ETS samples from the lung cancer cases, leukocytes represented approximately 10% till 50% of cells reflecting quite accurately the grade of inflammatory infiltration present on the histological sections. Moreover, a relatively high abundance of tumoral cells was detected on cytospin specimens with the Giemsa and cytokeratin stainings, ranging from 20% to 70% of cells. It is worth noting that besides tumor and inflammatory cells we observed "nude" cell nuclei, surrounded by sparse cytoplasmic remnants which did not stain with any of the used antibodies.

Such finding of uncertain cellular origin was detectable at different extent in almost all the examined samples. It is not easy to determine whether it derives from the sample preparation procedure or is an effect of the drying step of the samples on the microscope glasses after cytospinning. However, it is reasonable to assume that the mechanical and chemical stress which the tissue specimens are exposed to in order to obtain the single cell suspension may induce lysis of some tumor cells.

Finally, the presence of stromal components as determined by the SMA staining was restricted to 5% till at maximum of 20% of the cytospin content in the form of isolated irregular fibrillary fragments, demonstrating an effective removal of such components by the filtration of the tumor homogenates through the cell strainer.

**Figure 1:**
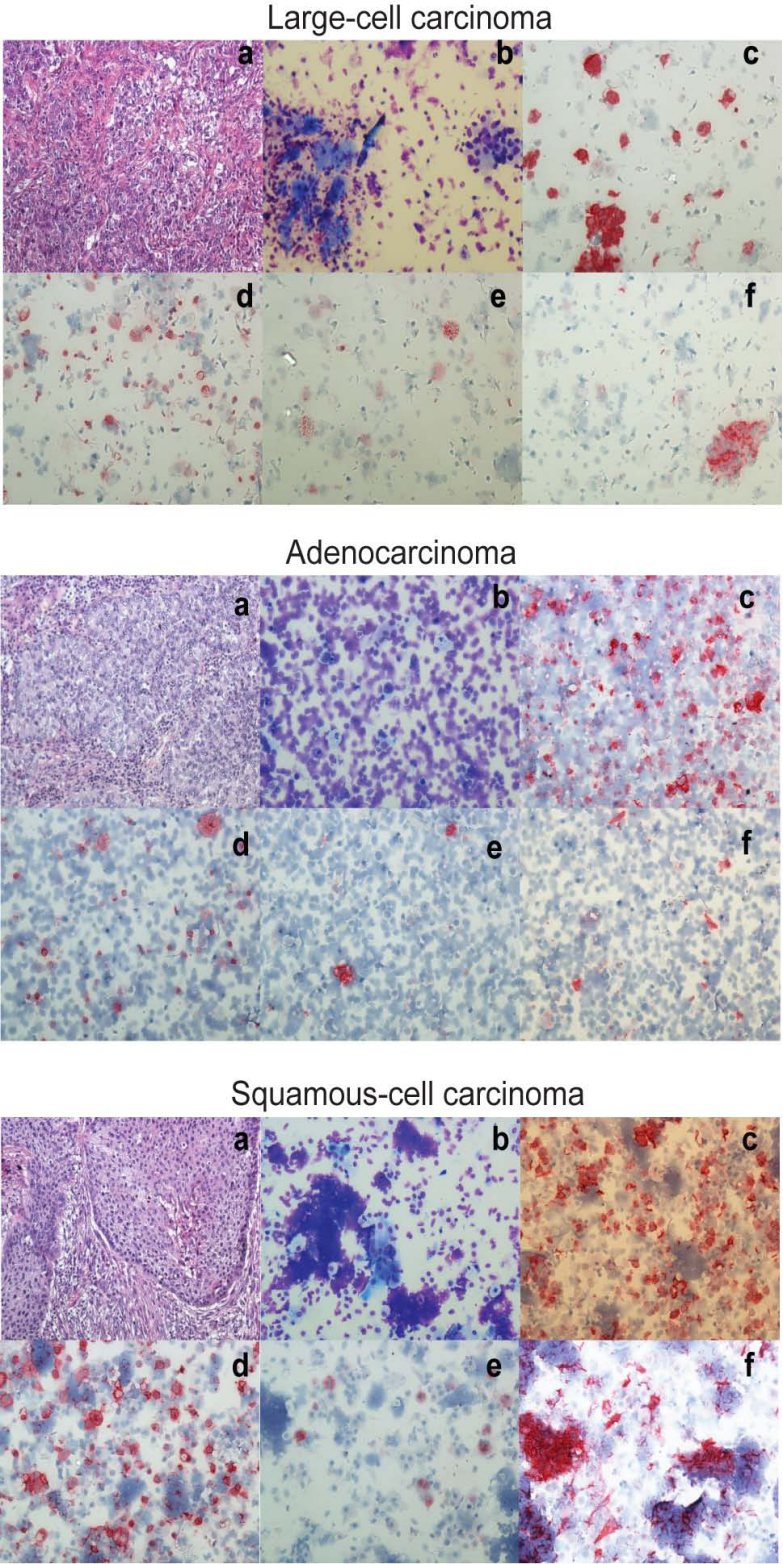
**Representative ICC stainings on cytospin specimens of lung large-cell carcinoma, adenocarcinoma and squamous-cell carcinoma**. a: histology (H&E); b: Giemsa; c: MNF116; d: CD45; e: CD68; f: SMA. (Magnification 200×)

Figure 1 depicts the stainings of representative cases of lung large-cell carcinoma, adenocarcinoma and squamous-cell carcinoma.

### Proteome analysis

To evaluate the applicability of the two protocols for proteomics studies both qualitative and quantitative mass spectrometry data was evaluated. Looking at the number of identified proteins a total number of 109 proteins was identified from the 'fresh frozen' preparation method and 244 from the cell suspension method (≥95% confidence cut off level) (Table 3). A list of all identified proteins from this study (≥95% confidence cut off level), and a list of all identified peptides, including coverage, score and iTRAQ error values can be found as supplementary material (supplementary file 1 and 2).

**Table 3:** Protein identification summary.

	# Identified proteins with ≥ 95% confidence	FDR	ppm
**ETS samples**	244	2.60%	62
**FF samples**	109	2.60%	62

(Abbreviations: ETS, enriched tumor cell suspension; FF, fresh-frozen; FDR, false discovery rate; ppm, part-per-million)

To calculate the reproducibility of the in-house developed ETS method the same tumor sample of lung adenocarcinoma was prepared in order to generate five replicates, as described above. In addition reproducibility was calculated for the mass spectrometry workflow alone, dividing one cell suspension sample in four parts and performing the precipitation, digestion, labeling etc separately. In general the CVs were very low, below 15% for the entire protocol including both sample preparation and MS analysis and below 10% for the MS workflow alone (table 4).

**Table 4:** Reproducibility of the sample preparation method.

	CV peptide level	CV protein level
**MS workflow**	9%	5%
**Sample preparation + MS workflow**	13%	7%

Coefficient of variance (CV) calculated based on all peptides and proteins ≥ 95% confidence.

**Figure 2:**
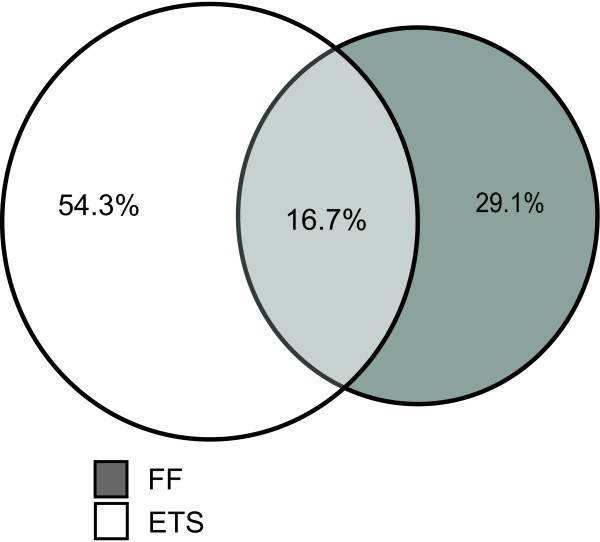
**Overlap of identified proteins between the two methods based on all proteins ≥ 95% confidence**.

**Figure 3:**
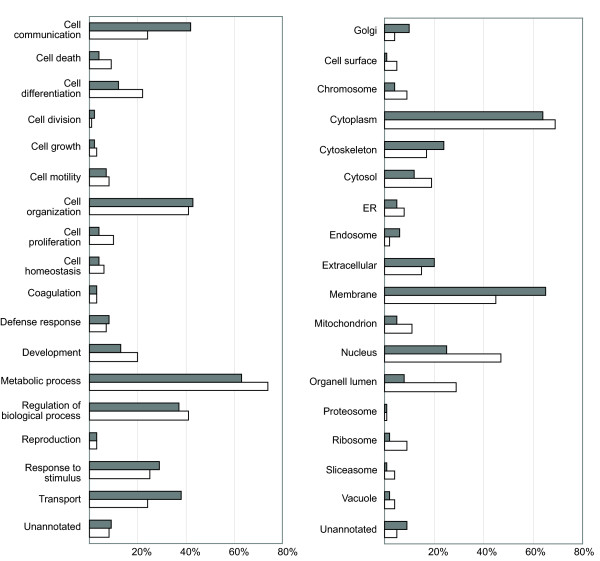
**Distribution of GO terms among the identified proteins**. Proteins (≥95% confidence) unique to the fresh frozen tissue lysis protocol (grey) and the enriched cell suspension (white). Percent of total number of proteins on the x-axis.

We analyzed the protein composition of the samples from the two different preparation methods in terms of overlap of all protein identifications and of analysis on frequency of gene ontology (GO) terms. The proportion of overlapping proteins was relatively small, with only 16.7% of all identified proteins overlapped between the two methods (Figure 2). However, analyzing the frequency of GO terms assigned to proteins unique for each method the general protein composition of samples was similar (Figure 3). The differences that were found were as expected considering the comparison of direct tissue lysates versus cell suspension analysis, with a larger proportion of GO terms related to tissue function and structure in the proteins extracted from the FF samples such as; cell communication, cell organization, defense response, transport, extracellular proteins and membrane proteins. This was further confirmed when analyzing protein abundance, as estimated by protein sequence coverage, where the most abundant proteins from the tissue lysis protocol included proteins derived from blood such as hemoglobin and albumin (Table 5).

**Table 5:** Proteins with top ten highest sequence coverage from tissue lysis protocol and cell suspension protocol.

Enriched Tumor cell Suspension samples (ETS)		
%Coverage	Accession	Name
45	IPI00479145.2	KRT19 Keratin, type I cytoskeletal 19
45	IPI00719280.2	UBB;RPS27A;UBC ubiquitin B precursor
40	IPI00894498.1	ACTB Beta actin variant (Fragment)
34	IPI00795719.1	cDNA FLJ53570, highly similar to Keratin, type I cytoskeletal 16
34	IPI00641249.2	ATP5A1 18 kDa protein
32	IPI00789324.3	JUP cDNA FLJ60424, highly similar to Junction plakoglobin
32	IPI00396378.3	HNRNPA2B1 Isoform B1 of Heterogeneous nuclear ribonucleoproteins A2/B1
31	IPI00554648.3	KRT8 Keratin, type II cytoskeletal 8
30	IPI00747707.2	KRT17 Radiated keratinocyte mRNA 266
29	IPI00216952.1	LMNA Isoform C of Lamin-A/C

**Fresh-Frozen samples (FF)**		
**%Coverage**	**Accession**	**Name**

39	IPI00790892.1	ENO2 6 kDa protein
36	IPI00796636.14	HBB Hemoglobin (Fragment)
28	IPI00027462.1	S100A9 Protein S100-A9
27	IPI00217473.5	HBZ Hemoglobin subunit zeta
24	IPI00745872.2	ALB Isoform 1 of Serum albumin
22	IPI00910407.1	cDNA FLJ53060, moderately similar to Peptidyl-prolyl cis-trans isomerase A
22	IPI00030929.4	MYL9 myosin regulatory light chain 9 isoform b
21	IPI00894498.1	ACTB Beta actin variant (Fragment)
21	IPI00903243.1	LOC284100 cDNA FLJ37577 fis, clone BRCOC2003513
20	IPI00747707.2	KRT17 Radiated keratinocyte mRNA 266

To evaluate the applicability of the protocols for comparative proteomics studies the quantitative differences between the normal tissue and the individual histology subtypes were explored. Comparing the normal tissue and the tumor tissue in the two different preparation protocols it was evident that there was a large contamination of blood in the normal tissue prepared with the total tissue lysis of fresh frozen samples. Both hemoglobin and albumin were identified as significantly (p ≤ 0.05) up-regulated in the fresh frozen lysates of normal samples compared with the tumor samples. This phenomenon could not be seen in the cell suspension samples. In addition, we observed differential quantitative expression of several markers according to histological subtype. For instance, we found that desmoplakin, which has been reported to be up-regulated in squamous-cell carcinoma [10] was found to be significantly (p ≤ 0.05) over expressed in the ETS of squamous-cell carcinoma samples, compared with adenocarcinomas and large-cell carcinomas. Similarly, up-regulated markers for adenocarcinoma (galectin-3-binding protein [11]) and large-cell carcinoma (S100A8 and S100A9 [12]) were also detected. Representative iTRAQ spectra of one of S100A8 and S100A9 peptides, respectively, depicting the differential relative abundance of these peptides between the four sample types can be found as supplementary file 3.

## Discussion

In this study we present a sample preparation method for mass spectrometry based proteomics analysis of lung cancer specimens. Our strategy aims at obtaining an enriched tumor cell suspension by removing the stromal and blood/plasma components through filtration and sequential washings, avoiding lysis of the majority of tumor cells. We show that, with our preparation method we are able to identify twice as many proteins compared with the direct lysis of fresh frozen samples. This is probably ascribed to the contamination by high abundant blood and plasma proteins present in the FF samples, which may have had a significant confounding effect on the entire proteomics experiment, limiting the possibilities to identify tumor specific proteins. Removal of plasma and red blood cell components was particularly efficient in the ETS preparation. In fact, albumin and hemoglobin were among the top ten most abundant proteins in the fresh frozen lysates, whereas in the ETS preparations albumin was ranked 33^*rd *^by sequence coverage-based ranking and hemoglobin was not identified at all.

In terms of cell content, tumor cells accounted for 20-70% of the cells in the ETS, with the remaining cell population being mainly inflammatory cells. Leukocytes cannot be removed by the filtration step in the protocol, as they are smaller than the average NSCLC tumor cells. However, the high variation in cell content detected on the cytospin specimens relatively accurately reflected the tissue composition of the original tissue samples, as shown by the comparison of the histology slides with the cytological samples from the same tumors.

Apart from the shown improvement in outcome of the proteomics analyses, the reproducibility of this method was shown to be very good, with coefficient of variance <15%. Additional advantages of this sample preparation method are that it is feasible, cheap and easy to conduct, not needing dedicated equipment.

As previously mentioned, most investigators performing gel- or MS-based proteomics analyses on lung cancer tissue samples use to obtain a direct lysis of fresh-frozen archival samples sometimes in combination with various forms of mechanical mincing [2-6]. Another strategy has been to perform macrodissection of frozen samples, with sequential controls of freeze-cut sections, to obtain a concentration of at least 70% of tumor cells [13,14]. Although this method aims at mainly removing stromal and vascular components it does not remove high abundant plasma proteins.

The most targeted method to enable a separate analysis of tumor cells and tumoral stromal components is laser capture microdissection (LCM) [15-17]. However LCM is time consuming, needs proper equipment and compared to our method the final recovery of tumor material is much lower and may not be sufficient for multiple analyses. In addition, the limited amount of material obtained from LCM compromises the potential of using extensive pre-fractionation often needed for large proteome coverage in gel- and MS-based proteomics workflows.

In order to expand the perspectives of proteomics studies by targeting larger patient cohorts and more specific and detailed clinical questions, there has recently been great interest regarding extraction of proteins from archival formalin-fixed paraffin embedded (FFPE) samples. A couple of studies on colon cancer and glioblastoma have reported comparable outcome of shotgun proteomics, in terms of number and class of identified proteins, on extracts from frozen or FFPE samples from the same patients, with or without the aid of LCM [18,19]. FFPE samples have the great advantage that they are being routinely collected in the clinical setting, in general are available for research and allow the selection of clinical cohorts with long follow up. In addition, cell morphology is usually highly conserved in FFPE specimens. However, it is not clear to what extent storage conditions and storage time, which can extend over even decades, and sample handling from surgical resection till fixation, may influence protein composition, especially in terms of integrity of post-translational modifications.

Finally, an alternative analytical strategy has been to directly place frozen tissue sections on a MALDI plate and analyze protein expression by so-called MALDI imaging [20-22]. This method requires very little starting material and has been implemented to discover lung cancer specific or lung cancer prognostic proteomics signatures, on the basis of the intensity of the diverse MS peaks. Nevertheless, since the identity of such proteins has not yet been reported, it still remains unclear whether the interesting peaks correspond to specific tumor biomarkers or represent high abundant proteins expressed at various levels.

Our sample preparation method aims at obtaining a compromise between the methods mentioned above, in terms of protein recovery and specificity of analyzed cells. However, a number of limitations must be acknowledged.

Firstly, fresh tumor material, as opposed to frozen or FFPE specimens, likely is a pre-requisite to obtain good results using this method. To be efficiently set up, the upstream logistics of this workflow need an accurate and constant collaboration between surgeons, pathologists and the proteomics lab. On the other hand, none of our results suggests that the procedure would not function on archival frozen samples if these are being collected directly after surgical resection and properly stored. In fact, we have previously demonstrated good subcellular enrichment of the nuclear protein component from fresh frozen tumor material [23]. Certainly, a clear disadvantage of our method is that the starting material must derive from surgical resection of entire tumors. Small biopsies obtained by needle aspiration would not be suitable samples to prepare with this method, since a lot of material will likely get lost during the diverse passages and washing steps of the protocol. The availability of adequate tumor material for research analyses is a general problem when dealing with lung cancer and this is one of the reasons why we excluded SCLC from our analysis. In fact, while at least 25% of patients with NSCLC receive a curative surgical resection as many as 90% of SCLC cases have already spread to the mediastinum when the disease is diagnosed. Hence it is technically and oncologically inoperable, and the only available tumor material consists of small biopsies obtained with minimally invasive procedures.

Another limitation of the present study is that we cannot produce evidence supporting the use of the described sample preparation method on tumor types other than NSCLC. The outcome may very well vary depending on the histological composition and the architectural structure of the diverse tumor classes, in terms of necrotic areas, vascularity and mucinous stromal components.

In addition we were not able to obtain pure tumor cell suspensions, since the samples were contaminated by various degrees of inflammatory cells. As a general consideration, the grade of inflammatory infiltration, as well as the relative amount of necrotic areas, must be taken into account as additional information when performing biomarker analysis by quantitatively comparing protein expression between diverse tumor samples, to avoid the risk of false positive results related to proteins not directly derived from the tumor cells. A number of multivariate data analysis methods can be used to determine which variables contribute to build significant models to answer the clinical question, and be hence qualified as potential biomarkers, and to avoid the generation of predictive models correlating with the grade of inflammation/necrosis on tissue specimens. Moreover, candidate biomarkers emerging from the proteomics experiments need subsequent validation on tissue by means of established antibody-based methods, such as immonohistochemistry, which will be used to assess the exact localization, expression and origin of the identified proteins and confirm their clinical role.

An alternative strategy to overcome at the experimental level the contamination by inflammatory cells could be to add a separation step for example with the aid of antibody-coated magnetic beads targeting immunological or epithelial cells. A positive isolation of tumor cells has been successfully implemented in pancreatic cancer samples using the surface molecule Ep-Cam as antigen [24]. This protein is also expressed in the majority of lung cancer cases and could be a suitable target. With the same technique, white blood cells could be removed by negative selection, however, at present it is unclear as to how many and which antigens should be used for efficient removal of this component. Moreover, the additional cell removal step in either the positive or negative form would require extensive testing of antibodies and optimal conditions and would hence qualify for a further development of the here presented method.

Finally, we have shown that our sample preparation workflow, optimized for tumor tissue, was not effective to obtain enrichment of bronchial and alveolar cells from normal lung tissue, although the removal of blood and plasma components was highly efficient compared to the direct tissue lysis. Proteomics analyses of lysates from fresh frozen samples of lung parenchyma to discover biomarkers of pulmonary diseases have been reported in a couple of studies [25,26]. Interest in implementing proteomics technologies to explore normal lung is rising, highlighting the need to further develop a preparation protocol specifically dedicated to extract proteins from normal lung cells.

## Conclusions

In summary, we present a method to reduce extracellular protein contamination in fresh NSCLC specimens for proteomics experiments. MS-based proteomics on samples prepared with the described protocol show that the efficient removal of primarily red blood cells and of high abundant plasma proteins allow the identification of a higher number of proteins and broader proteome coverage, compared to the crude lysis of fresh frozen archival samples.

The sample preparation method is feasible and reproducible and can be successfully implemented to perform biomarker discovery on NSCLC tissue samples with in-depth proteomics analysis.

## Competing interests

The authors declare that they have no competing interests.

## Authors' contributions

JL, LDP and MP designed and interpreted the study. LDP conducted the sample preparation. MP performed the proteomics experiments and the MS data analysis. GE and LDP evaluated the pathological and cytological specimens. JL and RL were scientific leads and participated in the design of the study. PB and LO provided the samples and contributed in establishing the sample collection workflow. LDP, JL and MP wrote the manuscript. All authors read and approved the final manuscript.

## Supplementary Material

Additional file 1 **List of all identified proteins**. the file contains the list of all proteins identified by MS/MS analyses of the lysates from the ETS and FF samples. Click here for file

Additional file 2 **List of all identified peptides**. the file contains the list of all peptides identified by MS/MS analyses of the lysates from the ETS and FF samples. Click here for file

Additional file 3 **Representative iTRAQ spectra**. Representative iTRAQ spectra of one of the peptides from the proteins S100A8 and S100A9 identified by MS/MS analysis of lysates from the ETS preparation showing a higher relative abundance of these proteins in the samples of large-cell tumors compared with the other histological types and with the normal lung samples. Click here for file
