# Supplementary material for: A novel method for sample preparation of fresh lung cancer tissue for proteomics analysis by tumor cell enrichment and removal of blood contaminants
Source: Proteome Sci. 2010 Feb 26;8:9. doi: 10.1186/1477-5956-8-9 (PMC2847553; doi:10.1186/1477-5956-8-9)
Supplement: Additional file 1 — List of all identified proteins. the file contains the list of all proteins identified by MS/MS analyses of the lysates from the ETS and FF samples. [file 1477-5956-8-9-S1.PDF]

Fresh frozen

N	%Cov	Accession	Name
1	23.97372723	IPI00745872,2	ALB Isoform 1 of Serum albumin
1	21.21212184	IPI00022434,4	ALB Putative uncharacterized protein ALB
2	19.77077425	IPI00747707,2	KRT17 Radiated keratinocyte mRNA 266
2	15.97222239	IPI00450768,7	KRT17 Keratin, type I cytoskeletal 17
3	16.40211642	IPI00796776,1	KRT5 cDNA FLJ54081, highly similar to Keratin, type II cytoskeletal 5
3	15.76271206	IPI00009867,3	KRT5 Keratin, type II cytoskeletal 5
4	22.79202342	IPI00894365,2	ACTB cDNA FLJ52842, highly similar to Actin, cytoplasmic 1
4	21.33333385	IPI00894498,1	ACTB Beta actin variant (Fragment)
4	21.33333385	IPI00021440,1	ACTG1 Actin, cytoplasmic 2
4	21.33333385	IPI00021439,1	ACTB Actin, cytoplasmic 1
5	14.37768191	IPI00418471,6	VIM Vimentin
6	18.96024495	IPI00916818,1	- cDNA FLJ53125, highly similar to Phosphoglycerate kinase 1
6	17.02637821	IPI00219568,4	PGK2 Phosphoglycerate kinase 2
6	16.30695462	IPI00169383,3	PGK1 Phosphoglycerate kinase 1
7	18.30985844	IPI00013991,1	TPM2 Isoform 1 of Tropomyosin beta chain
7	17.60563403	IPI00220709,3	TPM2 Isoform 2 of Tropomyosin beta chain
7	13.38028163	IPI00646748,1	TPM2 Tropomyosin 2
7	11.80124208	IPI00513698,1	TPM2 Tropomyosin 2
8	13.27433586	IPI00455315,4	ANXA2 Annexin A2
8	12.60504276	IPI00418169,3	ANXA2 annexin A2 isoform 1
8	11.04972363	IPI00903334,1	- cDNA FLJ34687 fis, clone MESAN2000620, highly similar to Annexin A2
8	8.849557489	IPI00334627,3	ANXA2P2 Putative annexin A2-like protein
9	39.21568692	IPI00790892,1	ENO2 6 kDa protein
9	7.373271883	IPI00465248,5	ENO1 Isoform alpha-enolase of Alpha-enolase
9	7.326007634	IPI00909595,1	- cDNA FLJ53099, highly similar to Beta-enolase
9	5.911330134	IPI00909949,1	- cDNA FLJ53926, highly similar to Beta-enolase
9	5.529953912	IPI00218474,5	ENO3 Beta-enolase
9	4.608295113	IPI00216171,3	ENO2 Gamma-enolase
10	28.07017565	IPI00027462,1	S100A9 Protein S100-A9
11	3.298810869	IPI00644576,1	FLNA Filamin A, alpha
11	3.258810192	IPI00302592,2	FLNA Isoform 2 of Filamin-A

11	3.24896127	IPI00333541,6	FLNA Isoform 1 of Filamin-A
11	2.980561554	IPI00909642,1	- Filamin A
12	10.43956056	IPI00465439,5	ALDOA Fructose-bisphosphate aldolase A
12	9.090909362	IPI00796333,1	ALDOA 45 kDa protein
13	10.23622081	IPI00910438,1	SND1 cDNA FLJ54574, highly similar to Staphylococcal nuclease domain-containing protein 1
13	10.00000015	IPI00140420,4	SND1 Staphylococcal nuclease domain-containing protein 1
14	36.19047701	IPI00796636,14	HBB Hemoglobin (Fragment)
14	25.85034072	IPI00654755,3	HBB Hemoglobin subunit beta
14	23.80952388	IPI00829896,1	HBD Hemoglobin Lepore-Baltimore (Fragment)
14	21.71428502	IPI00884107,1	HBB Beta-globin gene from a thalassemia patient
15	10.9302327	IPI00554788,5	KRT18 Keratin, type I cytoskeletal 18
16	8.333333582	IPI00785067,1	IGH@ IGH@ protein
16	7.599999756	IPI00423461,3	IGHA2 Putative uncharacterized protein DKFZp686C02220 (Fragment)
16	7.429718971	IPI00894384,1	IGHA2 Putative uncharacterized protein DKFZp686O16217 (Fragment)
17	3.730017692	IPI00742780,1	MYH9 FLJ00279 protein (Fragment)
17	2.653061226	IPI00019502,3	MYH9 Isoform 1 of Myosin-9
17	2.170767076	IPI00395772,5	MYH9 Isoform 2 of Myosin-9
18	2.062187903	IPI00918002,1	MUC5AC Mucin 5AC, oligomeric mucus/gel-forming
18	1.960104145	IPI00855918,1	MUC5B mucin 5, subtype B, tracheobronchial
18	1.595651358	IPI00886990,1	MUC5B Mucin-5B
19	7.633587718	IPI00003362,2	HSPA5 HSPA5 protein
20	22.48062044	IPI00910407,1	- cDNA FLJ53060, moderately similar to Peptidyl-prolyl cis-trans isomerase A
20	17.68292636	IPI00741973,1	LOC131691 similar to peptidyl-Pro cis trans isomerase
20	17.57575721	IPI00419585,9	PPIA Peptidyl-prolyl cis-trans isomerase A
20	14.5161286	IPI00376170,4	LOC390956 similar to peptidyl-Pro cis trans isomerase
20	13.00448477	IPI00887678,1	LOC654188 similar to peptidylprolyl isomerase A-like
20	12.85714358	IPI00888100,1	LOC390956 similar to peptidylprolyl isomerase A-like
20	10.40462404	IPI00787316,3	- Putative uncharacterized protein
21	15.42288512	IPI00216138,6	TAGLN Transgelin
22	9.090909362	IPI00909140,1	- cDNA FLJ53012, highly similar to Tubulin beta-7 chain
22	5.164319277	IPI00645452,1	TUBB Tubulin, beta polypeptide
22	4.954954982	IPI00011654,2	TUBB Tubulin beta chain
22	4.36681211	IPI00848150,1	LOC92755 Similar to Tubulin beta1

22	3.785489127	IPI00908770,1	- cDNA FLJ53063, highly similar to Tubulin beta-7 chain
22	2.762430906	IPI00910742,1	- cDNA FLJ53341, highly similar to Tubulin beta-4 chain
22	2.688172087	IPI00647896,1	TUBB Tubulin, beta
22	2.645502612	IPI00640115,1	TUBB3 HCG1983504, isoform CRA_f
22	2.624671906	IPI00643158,1	TUBB6 43 kDa protein
22	2.444987744	IPI00641706,1	TUBB6 46 kDa protein
22	2.252252214	IPI00023598,2	TUBB4 Tubulin beta-4 chain
22	2.247191034	IPI00031370,3	TUBB2B Tubulin beta-2B chain
22	2.247191034	IPI00013475,1	TUBB2A Tubulin beta-2A chain
22	2.237136476	IPI00646779,2	TUBB6 TUBB6 protein
22	2.222222276	IPI00013683,2	TUBB3 Tubulin beta-3 chain
22	1.254705153	IPI00152453,1	TUBB3 HCG2042771
23	15.76576531	IPI00232898,2	RAB41 RAB41, member RAS oncogene family
23	15.0289014	IPI00740750,2	- Putative uncharacterized protein ENSP00000331047 (Fragment)
23	14.00966197	IPI00024282,1	RAB8B Ras-related protein Rab-8B
23	13.58024627	IPI00903040,1	- cDNA FLJ38125 fis, clone D6OST2000127, moderately similar to RAS-RELATED PROTEIN RAB-8B
23	12.86549717	IPI00794226,1	RAB34 19 kDa protein
23	12.79069781	IPI00789395,1	RAB43 9 kDa protein
23	12.5	IPI00643312,1	RAB27B 10 kDa protein
23	11.95652187	IPI00790414,1	RAB8B 10 kDa protein
23	11.73708886	IPI00477489,1	RAB4B Isoform 1 of Ras-related protein Rab-4B
23	10.42653993	IPI00796900,1	RAB34 24 kDa protein
23	10.42653993	IPI00794306,1	RAB34 Ras-related GTP-binding protein RAB39
23	10.08064523	IPI00187143,1	RAB4B Isoform 2 of Ras-related protein Rab-4B
23	10.00000015	IPI00735546,2	LOC651324 similar to RAB1, member RAS oncogene family
23	9.931506962	IPI00910170,1	- cDNA FLJ59582, highly similar to Ras-related protein Rab-3A
23	9.777777642	IPI00795399,1	RAB34 25 kDa protein
23	9.499999881	IPI00871366,1	RAB1B Small GTP-binding protein
23	9.452736378	IPI00896425,1	RAB1B RAB1B protein
23	9.452736378	IPI00374519,3	RAB1C Putative Ras-related protein Rab-1C
23	9.452736378	IPI00008964,3	RAB1B Ras-related protein Rab-1B
23	9.401709586	IPI00790959,2	RAB34 RAB39 isoform 3
23	9.282700717	IPI00908406,1	- cDNA FLJ54253, highly similar to Ras-related protein Rab-34

23	9.268292785	IPI00873902,1	RAB1A 23 kDa protein
23	9.268292785	IPI00005719,1	RAB1A Isoform 1 of Ras-related protein Rab-1A
23	9.090909362	IPI00448725,1	RAB4B HCG1995540, isoform CRA_b
23	8.839779347	IPI00646415,1	RAB14 20 kDa protein
23	8.7649405	IPI00410673,1	RAB34 Isoform 2 of Ras-related protein Rab-34
23	8.730158955	IPI00797402,1	RAB34 Putative uncharacterized protein RAB34
23	8.494208753	IPI00328180,4	RAB34 Isoform 1 of Ras-related protein Rab-34
23	8.461538702	IPI00791799,1	RAB34 29 kDa protein
23	8.18181783	IPI00023504,1	RAB3A Ras-related protein Rab-3A
23	8.029197156	IPI00788998,2	RAB34 RAB39 isoform 2
23	7.801418751	IPI00793155,1	RAB34 RAB39 isoform 1
23	7.500000298	IPI00016513,5	RAB10 Ras-related protein Rab-10
23	7.4626863	IPI00014603,1	RAB9B Ras-related protein Rab-9B
23	7.441860437	IPI00291928,8	RAB14 Ras-related protein Rab-14
23	7.236842066	IPI00556414,1	RAB8A Mel transforming oncogene variant (Fragment)
23	6.875000149	IPI00910398,1	- cDNA FLJ57794, moderately similar to Ras-related protein Rab-35
23	6.748466194	IPI00022134,1	RAB1B Putative small GTP-binding protein
23	6.707317382	IPI00795889,1	RAB34 18 kDa protein
23	6.707317382	IPI00425916,1	RAB30 Putative uncharacterized protein DKFZp686J07132
23	6.62650615	IPI00793203,1	RAB35 18 kDa protein
23	6.508875638	IPI00789993,1	RAB37 RAB37, member RAS oncogene family, isoform CRA_b
23	6.077348068	IPI00785140,3	- Putative Rab-43-like protein ENSP00000330714
23	6.043956056	IPI00788648,1	RAB37 cDNA FLJ45130 fis, clone BRAWH3037428, highly similar to Homo sapiens RAB37, member RAS oncogene
23	5.913978443	IPI00790499,1	RAB37 Putative uncharacterized protein RAB37
23	5.82010597	IPI00791421,1	RAB37 RAB37, member RAS oncogene family, isoform CRA_a
23	5.759162456	IPI00792992,1	RAB37 Putative uncharacterized protein RAB37
23	5.673758686	IPI00334174,3	RAB1A Isoform 2 of Ras-related protein Rab-1A
23	5.472636968	IPI00300096,4	RAB35 Ras-related protein Rab-35
23	5.418719351	IPI00302030,2	RAB30 Ras-related protein Rab-30
23	5.314009637	IPI00909683,1	- cDNA FLJ53028, highly similar to Ras-related protein Rab-8A
23	5.314009637	IPI00028481,1	RAB8A Ras-related protein Rab-8A
23	5.288461596	IPI00383449,2	RAB15 Isoform 2 of Ras-related protein Rab-15
23	5.288461596	IPI00217943,3	RAB6A Isoform 2 of Ras-related protein Rab-6A

23	5.288461596	IPI00023526,4	RAB6A Isoform 1 of Ras-related protein Rab-6A
23	5.288461596	IPI00016891,1	RAB6B Ras-related protein Rab-6B
23	5.213269964	IPI00886865,1	LOC100134367 similar to RAB12, member RAS oncogene family
23	5.188679323	IPI00394882,2	RAB15 Isoform 1 of Ras-related protein Rab-15
23	5.188679323	IPI00329441,1	RAB43 Ras-related protein Rab-43
23	5.164319277	IPI00219858,6	RAB27A Isoform Short of Ras-related protein Rab-27A
23	5.164319277	IPI00060801,1	RAB39B Ras-related protein Rab-39B
23	5.092592537	IPI00332970,2	RAB37 RAB37, member RAS oncogene family isoform 3
23	5.069124326	IPI00001618,2	RAB39 Ras-related protein Rab-39A
23	5.045871437	IPI00480056,8	RAB4A RAB4A, member RAS oncogene family variant
23	5.045871437	IPI00010491,3	RAB27B Ras-related protein Rab-27B
23	5.022830889	IPI00300562,2	RAB3B Ras-related protein Rab-3B
23	5.022830889	IPI00032808,1	RAB3D Ras-related protein Rab-3D
23	4.977375641	IPI00016381,2	RAB27A Isoform Long of Ras-related protein Rab-27A
23	4.932735488	IPI00073180,8	RAB37 Ras-related protein Rab-37
23	4.867256805	IPI00871276,1	RAB3C Putative uncharacterized protein RAB3C (Fragment)
23	4.845815152	IPI00061114,1	RAB3C Ras-related protein Rab-3C
23	4.824561253	IPI00798392,1	RAB37 Putative uncharacterized protein RAB37
23	4.803493619	IPI00021475,1	RAB33B Ras-related protein Rab-33B
23	4.651162773	IPI00884187,2	EFCAB4B cDNA FLJ61047, weakly similar to Homo sapiens RAS and EF-hand domain containing (RASEF), mRNA
23	4.624277353	IPI00917079,1	RAB1A 19 kDa protein
23	4.508196563	IPI00419932,5	RAB12 Putative Ras-related protein Rab-12
23	3.513513505	IPI00412443,1	RASEF Isoform 1 of RAS and EF-hand domain-containing protein
23	2.635542117	IPI00888647,1	FLJ43093 hypothetical protein LOC401258
23	2.635542117	IPI00888316,1	FLJ43093 similar to FLJ43093 protein
24	21.18644118	IPI00903243,1	LOC284100 cDNA FLJ37577 fis, clone BRCOC2003513, moderately similar to 14-3-3 protein epsilon
24	11.39896363	IPI00793344,1	YWHAE 22 kDa protein
24	8.627451211	IPI00000816,1	YWHAE 14-3-3 protein epsilon
24	8.365018666	IPI00086909,9	LOC440917 Similar to 14-3-3 protein epsilon
25	5.882352963	IPI00869004,1	SERPINA1 Isoform 3 of Alpha-1-antitrypsin
25	5.013927445	IPI00790784,2	SERPINA1 Isoform 2 of Alpha-1-antitrypsin
25	4.306220263	IPI00553177,1	SERPINA1 Isoform 1 of Alpha-1-antitrypsin
26	2.141434327	IPI00556632,3	FN1 Isoform 12 of Fibronectin

26	1.976102963	IPI00479723,3	FN1 Isoform 10 of Fibronectin
26	1.968864538	IPI00339226,1	FN1 Isoform 6 of Fibronectin
26	1.944821328	IPI00339225,1	FN1 Isoform 5 of Fibronectin
26	1.898454688	IPI00855777,1	FN1 Isoform 14 of Fibronectin
26	1.896779798	IPI00867588,1	FN1 Isoform 13 of Fibronectin
26	1.87282227	IPI00339228,1	FN1 Isoform 8 of Fibronectin
26	1.84549354	IPI00414283,5	FN1 fibronectin 1 isoform 4 preproprotein
26	1.825902425	IPI00339223,1	FN1 Isoform 3 of Fibronectin
26	1.802179404	IPI00339319,1	FN1 Isoform 11 of Fibronectin
26	1.802179404	IPI00022418,1	FN1 Isoform 1 of Fibronectin
26	1.801424474	IPI00873210,1	FN1 263 kDa protein
26	1.776125655	IPI00845263,1	FN1 fibronectin 1 isoform 2 preproprotein
26	1.757972129	IPI00339227,4	FN1 Isoform 7 of Fibronectin
26	1.735970937	IPI00855785,1	FN1 Isoform 15 of Fibronectin
26	1.033973414	IPI00339224,1	FN1 Isoform 4 of Fibronectin
27	18.00000072	IPI00387116,1	- Ig kappa chain V-III region NG9 (Fragment)
27	16.51376188	IPI00885193,1	IGKV3-20 Myosin-reactive immunoglobulin light chain variable region (Fragment)
27	16.51376188	IPI00827826,1	- Cold agglutinin FS-2 L-chain (Fragment)
27	16.51376188	IPI00387118,1	- Ig kappa chain V-III region WOL
27	16.51376188	IPI00387117,5	IGKV3D-20 Ig kappa chain V-III region Ti
27	16.51376188	IPI00387115,1	- Ig kappa chain V-III region SIE
27	16.51376188	IPI00385252,1	- Ig kappa chain V-III region GOL
27	14.70588297	IPI00916434,1	- Anti-(ED-B) scFV (Fragment)
27	14.70588297	IPI00915411,2	- Anti-(ED-B) scFV (Fragment)
27	13.95348907	IPI00384576,1	- Ig kappa chain V-III region HIC
27	13.95348907	IPI00030205,3	IGKV3-20 Ig kappa chain V-III region HAH
27	9.787233919	IPI00909649,1	IGKC IGKC protein
28	7.089715451	IPI00398002,6	PLEC1 Isoform 3 of Plectin-1
28	7.083515823	IPI00186711,4	PLEC1 Isoform 2 of Plectin-1
28	6.688815355	IPI00398776,3	PLEC1 plectin 1 isoform 7
28	6.674033403	IPI00420096,4	PLEC1 plectin 1 isoform 3
28	6.662254781	IPI00398775,3	PLEC1 plectin 1 isoform 2
28	6.641741842	IPI00398779,5	PLEC1 Isoform 4 of Plectin-1

28	6.641741842	IPI00398777,3	PLEC1 plectin 1 isoform 8
28	6.635904312	IPI00398778,3	PLEC1 plectin 1 isoform 10
28	6.447480619	IPI00014898,3	PLEC1 Isoform 1 of Plectin-1
28	5.186267197	IPI00887421,1	LOC652460 hypothetical protein, partial
29	9.638553858	IPI00793922,1	GAPDH 9 kDa protein
29	6.923077255	IPI00789134,1	GAPDH Glyceraldehyde 3-phosphate dehydrogenase
29	6.741572917	IPI00797221,7	GAPDH Glyceraldehyde-3-phosphate dehydrogenase
29	6.143344566	IPI00795257,1	GAPDH 32 kDa protein
29	5.373134464	IPI00219018,7	GAPDH Glyceraldehyde-3-phosphate dehydrogenase
29	4.972375557	IPI00788737,1	GAPDH 39 kDa protein
30	3.388746828	IPI00220216,1	TNC Isoform 6 of Tenascin
30	3.202416748	IPI00220214,1	TNC Isoform 5 of Tenascin
30	3.035509773	IPI00220211,1	TNC Isoform 2 of Tenascin
30	2.885138802	IPI00220212,1	TNC Isoform 3 of Tenascin
30	2.750389278	IPI00867560,1	- TNC protein
30	2.511848323	IPI00220213,1	TNC Isoform 4 of Tenascin
30	2.407996356	IPI00031008,1	TNC Isoform 1 of Tenascin
31	7.692307979	IPI00411633,4	HSP90AB1 Heat shock protein beta (Fragment)
31	4.972375557	IPI00414676,6	HSP90AB1 Heat shock protein HSP 90-beta
31	3.517587855	IPI00555614,1	HSP90AB3P Putative heat shock protein HSP 90-beta-3
32	7.748184353	IPI00908762,1	- cDNA FLJ53509, highly similar to Galectin-3-binding protein
32	6.597938389	IPI00902654,2	- cDNA FLJ54583, highly similar to Galectin-3-binding protein
32	5.776173249	IPI00887555,1	LOC100133842 similar to lectin, galactoside-binding, soluble, 3 binding protein
32	5.470085517	IPI00023673,1	LGALS3BP Galectin-3-binding protein
33	18.53932589	IPI00873994,1	KRT15 Putative uncharacterized protein KRT15 (Fragment)
33	15.38461596	IPI00908905,1	- cDNA FLJ58872, highly similar to Keratin, type I cytoskeletal 15
33	15.13157934	IPI00290077,2	KRT15 Keratin, type I cytoskeletal 15
33	12.85714358	IPI00873598,1	KRT13 46 kDa protein
33	12.85714358	IPI00171196,2	KRT13 keratin 13 isoform b
33	12.67605573	IPI00550661,2	KRT13 Isoform 2 of Keratin, type I cytoskeletal 13
33	11.79039329	IPI00009866,6	KRT13 Isoform 1 of Keratin, type I cytoskeletal 13
34	11.02803722	IPI00909059,1	- cDNA FLJ53910, highly similar to Keratin, type II cytoskeletal 6A
34	10.4609929	IPI00300725,7	KRT6A Keratin, type II cytoskeletal 6A

34	10.4609929	IPI00293665,8	KRT6B Keratin, type II cytoskeletal 6B
34	10.10638326	IPI00299145,9	KRT6C Keratin, type II cytoskeletal 6C
34	10.07604599	IPI00910738,1	- cDNA FLJ60647, highly similar to Keratin, type II cytoskeletal 6B
34	6.776859611	IPI00005859,4	KRT75 cDNA FLJ60809, highly similar to Homo sapiens cytokeratin type II (K6HF), mRNA
35	9.89761129	IPI00644497,4	IGHA1;IGHV3OR16-13 Hypothetical short protein
35	9.51417014	IPI00386879,1	IGHA1;IGHV3OR16-13 cDNA FLJ14473 fis, clone MAMMA1001080, highly similar to Homo sapiens SNC73 protein
35	9.330628812	IPI00430842,3	IGHA1;IGHV3OR16-13 IGHA1 protein
35	9.055876732	IPI00423462,5	IGHA1;IGHV3OR16-13 Putative uncharacterized protein DKFZp686K18196 (Fragment)
35	8.113590628	IPI00449920,1	IGHA1;IGHV3OR16-13 cDNA FLJ90170 fis, clone MAMMA1000370, highly similar to Ig alpha-1 chain C region
35	7.999999821	IPI00061977,1	IGHA1;IGHV3OR16-13 IGHA1 protein
35	7.28744939	IPI00647704,1	IGHA1;IGHV3OR16-13 cDNA FLJ41552 fis, clone COLON2004478, highly similar to Protein Tro alpha1 H,myeloma
35	7.214429229	IPI00166866,3	IGHA1;IGHV3OR16-13 IGHA1 protein
35	5.882352963	IPI00719233,1	IGHA1;IGHV3OR16-13 IGHA1 protein
35	5.846774206	IPI00744561,1	IGHA1;IGHV3OR16-13 IGHA1 protein
35	5.846774206	IPI00386524,3	IGHA1;IGHV3OR16-13 CDNA FLJ25298 fis, clone STM07683, highly similar to Protein Tro alpha1 H,myeloma
35	5.835010111	IPI00383164,1	IGHA1;IGHV3OR16-13 SNC66 protein
35	5.731225386	IPI00426060,3	IGHA1;IGHV3OR16-13 Putative uncharacterized protein DKFZp686J11235 (Fragment)
35	5.731225386	IPI00423460,3	IGHA1;IGHV3OR16-13 Putative uncharacterized protein DKFZp686G21220 (Fragment)
36	3.709949553	IPI00607778,1	MYH14 Isoform 4 of Myosin-14
36	3.585926816	IPI00029818,5	MYH14 Isoform 5 of Myosin-14
36	3.289473802	IPI00397526,3	MYH10 Isoform 1 of Myosin-10
36	3.26305218	IPI00479307,4	MYH10 Isoform 2 of Myosin-10
36	3.254882246	IPI00790503,3	MYH10 Isoform 3 of Myosin-10
36	2.945581637	IPI00337335,6	MYH14 Isoform 1 of Myosin-14
36	2.897838876	IPI00607818,2	MYH14 MYH14 variant protein
36	1.393188816	IPI00024870,1	MYH11 smooth muscle myosin heavy chain 11 isoform SM2A
36	1.388174761	IPI00744256,1	MYH11 smooth muscle myosin heavy chain 11 isoform SM2B
36	1.36916833	IPI00020501,1	MYH11 Myosin-11
36	1.364325453	IPI00873792,1	MYH11 228 kDa protein
36	1.364325453	IPI00743857,1	MYH11 smooth muscle myosin heavy chain 11 isoform SM1B
36	1.330704801	IPI00873982,2	MYH11 Myosin heavy chain 11 smooth muscle isoform
37	22.03389853	IPI00030929,4	MYL9 myosin regulatory light chain 9 isoform b
37	15.20467848	IPI00220573,4	MRCL3 Myosin regulatory light chain MRLC3

37	15.11627883	IPI00719669,4	MRLC2 Myosin regulatory light chain
37	15.11627883	IPI00033494,3	MRLC2 Myosin regulatory light chain MRLC2
37	15.11627883	IPI00220278,5	MYL9 Myosin regulatory light polypeptide 9
37	14.68926519	IPI00604523,1	MRCL3 Myosin regulatory light chain MRCL3 variant
37	8.695652336	IPI00642780,2	MRCL3 13 kDa protein
37	6.896551698	IPI00376572,2	LOC391722 similar to myosin:SUBUNIT=regulatory light chain
37	5.305039883	IPI00888438,1	LOC391722 similar to myosin:SUBUNIT=regulatory light chain
37	5.291005224	IPI00888129,1	LOC642076 similar to hCG1789038
38	1.586333103	IPI00455383,4	CLTC Isoform 2 of Clathrin heavy chain 1
38	1.552238781	IPI00024067,4	CLTC Isoform 1 of Clathrin heavy chain 1
39	7.95698911	IPI00784807,1	IGHG2 IGHG2 protein
39	7.888040692	IPI00830132,1	IGHG4 Putative uncharacterized protein IGHG4 (Fragment)
39	7.659574598	IPI00784810,1	IGHV4-31 IGHV4-31 protein
39	6.866952777	IPI00816314,1	IGHM Putative uncharacterized protein DKFZp686I15196
39	6.694560498	IPI00472610,2	IGHM IGHM protein
39	6.666667014	IPI00423463,1	IGHG1 Putative uncharacterized protein DKFZp686O01196
39	6.56779632	IPI00876888,1	- cDNA FLJ78387
39	6.060606241	IPI00736860,3	- Protein
39	5.68421036	IPI00423464,1	IGHG1 Putative uncharacterized protein DKFZp686K03196
39	5.416666716	IPI00761159,1	IGHM IGHM protein
39	5.263157934	IPI00784842,1	IGHV4-31 Putative uncharacterized protein DKFZp686G11190
39	5.089058354	IPI00829767,1	IGHG2 Protein
39	4.796162993	IPI00399007,5	IGHG2 Putative uncharacterized protein DKFZp686I04196 (Fragment)
39	4.477611929	IPI00829944,1	IGHG1 IGHG1 protein
39	4.449152574	IPI00645363,2	IGHG1 Putative uncharacterized protein DKFZp686P15220
39	4.421052709	IPI00807531,2	IGHG1 IGHG1 protein
39	4.365904257	IPI00423466,1	IGHG1 Putative uncharacterized protein DKFZp686H20196
39	4.356846586	IPI00384938,1	IGHG1 Putative uncharacterized protein DKFZp686N02209
39	4.281345382	IPI00829814,1	IGHG4 Ig gamma-4 chain C region
39	4.022988677	IPI00816681,1	IGHM Hepatitis B virus receptor binding protein (Fragment)
39	3.860294074	IPI00448925,3	IGHG1 IGHG1 protein
39	2.985074557	IPI00448938,1	IGHG1 IGHG1 protein
39	2.959830873	IPI00784822,1	IGHV4-31 IGHV4-31 protein

39	2.959830873	IPI00550640,2	IGHG4 IGHG4 protein
39	2.947368473	IPI00784817,1	IGHV4-31 Anti-RhD monoclonal T125 gamma1 heavy chain
39	2.941176482	IPI00785084,1	IGHV4-31 Immunoglobulin heavy variable 4-31
39	2.061855607	IPI00382606,1	F7 Factor VII active site mutant immunoconjugate
40	6.557376683	IPI00909247,1	- cDNA FLJ51655, highly similar to Actin-like protein 2
40	5.076142028	IPI00005159,3	ACTR2 Actin-related protein 2
40	5.012531206	IPI00749250,2	ACTR2 45 kDa protein
40	5.012531206	IPI00470573,1	ACTR2 actin-related protein 2 isoform a
41	17.29957759	IPI00793917,1	KRT8 27 kDa protein
41	8.488612622	IPI00554648,3	KRT8 Keratin, type II cytoskeletal 8
41	7.44920969	IPI00787323,2	hCG_1988300 Similar to Keratin, type II cytoskeletal 8
41	3.607214615	IPI00017870,1	- Keratin-8-like protein 1
42	16.73469394	IPI00021263,3	YWHAZ 14-3-3 protein zeta/delta
42	10.71428582	IPI00789337,2	YWHAZ cDNA, FLJ79516, highly similar to 14-3-3 protein zeta/delta
43	19.65811998	IPI00739205,3	LOC652102 Ig heavy chain V-I region HG3
43	19.49152499	IPI00829979,1	- Putative uncharacterized protein ENSP00000375008
43	19.16666627	IPI00895890,2	- Immunoglobulin mu-chain D-J4-region (Fragment)
43	19.16666627	IPI00888695,1	LOC100133862 similar to hCG1773549
43	19.16666627	IPI00419517,1	IGHV1-69 IGHV1-69 protein
43	8.870967478	IPI00384407,1	- Myosin-reactive immunoglobulin heavy chain variable region (Fragment)
43	8.870967478	IPI00382471,1	- Ig heavy chain V-I region WOL
43	3.235294297	IPI00887113,1	LOC642131 similar to hCG1812074
43	2.365591377	IPI00887332,1	LOC642131 similar to hCG1812074
44	6.217616424	IPI00908605,1	- cDNA FLJ59940, highly similar to Tubulin beta-2C chain
44	4.943820089	IPI00007752,1	TUBB2C Tubulin beta-2C chain
44	3.022670001	IPI00911016,1	- cDNA FLJ11352 fis, clone HEMBA1000020, highly similar to Tubulin beta-2C chain
45	8.074533939	IPI00220327,3	KRT1 Keratin, type II cytoskeletal 1
46	3.258655965	IPI00298497,3	FGB Fibrinogen beta chain
47	5.64263314	IPI00872379,1	ANXA5 Putative uncharacterized protein ANXA5 (Fragment)
47	5.624999851	IPI00329801,12	ANXA5 Annexin A5
48	2.912239358	IPI00298994,6	TLN1 Talin-1
49	12.305516	IPI00010740,1	SFPQ Isoform Long of Splicing factor, proline- and glutamine-rich
49	10.91180891	IPI00216613,1	SFPQ Isoform Short of Splicing factor, proline- and glutamine-rich

50	7.94871822	IPI00739237,1	LOC653879 similar to complement component 3
50	2.705953084	IPI00783987,2	C3 Complement C3 (Fragment)
51	9.352517873	IPI00020599,1	CALR Calreticulin
52	8.612440526	IPI00010471,5	LCP1 Plastin-2
52	3.252032399	IPI00646259,1	LCP1 Lymphocyte cytosolic protein 1
52	1.226993836	IPI00895834,1rev	BANK1 Isoform 4 of B-cell scaffold protein with ankyrin repeats
52	1.059602667	IPI00787434,1rev	BANK1 Isoform 3 of B-cell scaffold protein with ankyrin repeats
52	1.038961019	IPI00787141,1rev	BANK1 Isoform 2 of B-cell scaffold protein with ankyrin repeats
52	1.019108295	IPI00179337,5rev	BANK1 Isoform 1 of B-cell scaffold protein with ankyrin repeats
53	4.576271027	IPI00219750,1	CTNND1 Isoform 4 of Catenin delta-1
53	4.53020148	IPI00219749,1	CTNND1 Isoform 4C of Catenin delta-1
53	4.426229373	IPI00219747,1	CTNND1 Isoform 4A of Catenin delta-1
53	4.383116961	IPI00219745,1	CTNND1 Isoform 4AC of Catenin delta-1
53	4.361873865	IPI00219748,1	CTNND1 Isoform 4B of Catenin delta-1
53	4.320000112	IPI00219746,1	CTNND1 Isoform 4BC of Catenin delta-1
53	4.225352034	IPI00219744,1	CTNND1 Isoform 4AB of Catenin delta-1
53	4.186046496	IPI00219743,1	CTNND1 Isoform 4ABC of Catenin delta-1
53	3.620873392	IPI00848265,1	CTNND1 Putative uncharacterized protein DKFZp781O2021
53	3.325122967	IPI00219742,1	CTNND1 Isoform 3 of Catenin delta-1
53	3.300733492	IPI00219741,1	CTNND1 Isoform 3C of Catenin delta-1
53	3.245192394	IPI00219738,1	CTNND1 Isoform 3A of Catenin delta-1
53	3.221957013	IPI00219735,1	CTNND1 Isoform 3AC of Catenin delta-1
53	3.214285895	IPI00845246,1	CTNND1 catenin, delta 1 isoform 3B
53	3.210463747	IPI00219739,1	CTNND1 Isoform 3B of Catenin delta-1
53	3.187721223	IPI00219737,1	CTNND1 Isoform 3BC of Catenin delta-1
53	3.143189847	IPI00219732,1	CTNND1 Isoform 2 of Catenin delta-1
53	3.135888651	IPI00219734,1	CTNND1 Isoform 3AB of Catenin delta-1
53	3.121387213	IPI00219731,1	CTNND1 Isoform 2C of Catenin delta-1
53	3.114186786	IPI00219733,1	CTNND1 Isoform 3ABC of Catenin delta-1
53	3.071672283	IPI00219728,1	CTNND1 Isoform 2A of Catenin delta-1
53	3.050847538	IPI00219726,1	CTNND1 Isoform 2AC of Catenin delta-1
53	3.040540591	IPI00219730,1	CTNND1 Isoform 2B of Catenin delta-1
53	3.020134196	IPI00219727,1	CTNND1 Isoform 2BC of Catenin delta-1

53	2.973568253	IPI00219725,1	CTNND1 Isoform 2AB of Catenin delta-1
53	2.957283705	IPI00845519,1	CTNND1 Isoform 1 of Catenin delta-1
53	2.954048105	IPI00219875,1	CTNND1 Isoform 2ABC of Catenin delta-1
53	2.937976085	IPI00219873,1	CTNND1 Isoform 1C of Catenin delta-1
53	2.893890627	IPI00219870,1	CTNND1 Isoform 1A of Catenin delta-1
53	2.875399403	IPI00219868,1	CTNND1 Isoform 1AC of Catenin delta-1
53	2.866242081	IPI00219872,1	CTNND1 Isoform 1B of Catenin delta-1
53	2.848101221	IPI00219869,1	CTNND1 Isoform 1BC of Catenin delta-1
53	2.845100127	IPI00419482,3	CTNND1 catenin, delta 1 isoform 1B
53	2.803738229	IPI00182469,3	CTNND1 Isoform 1AB of Catenin delta-1
53	2.786377631	IPI00182540,5	CTNND1 Isoform 1ABC of Catenin delta-1
54	8.381503075	IPI00218918,5	ANXA1 Annexin A1
54	8.13953504	IPI00908577,1	- cDNA FLJ51887, highly similar to Annexin A1
55	5.521472543	IPI00910360,1	- cDNA FLJ58927, highly similar to Endoplasmin
55	2.615193091	IPI00027230,3	HSP90B1 Endoplasmin
56	9.941520542	IPI00879084,1	CP 20 kDa protein
56	3.286384791	IPI00017601,1	CP Ceruloplasmin
57	14.28571492	IPI00909303,1	- cDNA FLJ58073, moderately similar to Cathepsin B
57	14.13043439	IPI00903045,1	- cDNA FLJ40065 fis, clone TESOP2000400, highly similar to CATHEPSIN B
57	11.50442511	IPI00295741,4	CTSB Cathepsin B
58	15.42288512	IPI00884926,1	ORM1 orosomucoid 1 precursor
58	15.42288512	IPI00022429,3	ORM1 Alpha-1-acid glycoprotein 1
59	8.974359185	IPI00916391,1	TUBA4A 18 kDa protein
59	7.142857462	IPI00335314,3	TUBA4A Putative uncharacterized protein TUBA4A
59	5.809128657	IPI00017454,4	TUBA4B Putative tubulin-like protein alpha-4B
59	5.447470769	IPI00478908,3	TUBA1C 29 kDa protein
59	4.387990758	IPI00794663,1	TUBA4A Tubulin, alpha 1 (Testis specific), isoform CRA_a
59	4.294478521	IPI00784332,2	TUBA3C;TUBA3D Tubulin alpha-2 chain
59	4.241071269	IPI00007750,1	TUBA4A Tubulin alpha-4A chain
59	3.655352443	IPI00792478,1	TUBA8 Tubulin, alpha 8, isoform CRA_b
59	3.373493999	IPI00915791,1	TUBA3C;TUBA3D 46 kDa protein
59	3.365384787	IPI00792677,1	TUBA1B cDNA FLJ60097, highly similar to Tubulin alpha-ubiquitous chain
59	3.34928222	IPI00218345,5	TUBA3C;TUBA3D Isoform 2 of Tubulin alpha-3C/D chain

59	3.11804004	IPI00646909,2	TUBA8 Tubulin alpha-8 chain
59	3.11804004	IPI00218343,4	TUBA1C Tubulin alpha-1C chain
59	3.111111186	IPI00410402,3	TUBA3C;TUBA3D;TUBA3E Tubulin alpha-3E chain
59	3.111111186	IPI00179709,4	TUBA3C;TUBA3D Isoform 1 of Tubulin alpha-3C/D chain
59	3.10421288	IPI00387144,4	TUBA1B Tubulin alpha-1B chain
59	3.10421288	IPI00180675,4	TUBA1A Tubulin alpha-1A chain
59	2.997858636	IPI00791613,1	TUBA8 Putative uncharacterized protein DKFZp686L04275
60	7.818929851	IPI00787265,1	LOC729034 similar to puromycin sensitive aminopeptidase
60	5.078125	IPI00888617,1	LOC100132544 similar to puromycin sensitive aminopeptidase
60	2.719665319	IPI00455333,4	LOC440434 Isoform 1 of Puromycin-sensitive aminopeptidase-like protein
60	2.176278643	IPI00026216,4	NPEPPS Puromycin-sensitive aminopeptidase
60	2.073365264	IPI00902884,1	LOC440434 cDNA FLJ38558 fis, clone HCHON2003327, highly similar to Puromycin-sensitive aminopeptidase
61	3.831417486	IPI00789185,2	PYGB Phosphorylase
61	2.833530121	IPI00783313,2	PYGL Glycogen phosphorylase, liver form
61	1.344537828	IPI00909513,1	- cDNA FLJ55360, highly similar to Glycogen phosphorylase, muscle form
61	1.186239626	IPI00004358,4	PYGB Glycogen phosphorylase, brain form
61	1.061007939	IPI00657751,1	PYGM Phosphorylase
61	0.950118806	IPI00218130,3	PYGM Glycogen phosphorylase, muscle form
62	8.992806077	IPI00869068,1	LOC440396 Similar to Heterogeneous nuclear ribonucleoprotein A1
62	8.275862038	IPI00797902,1	HNRNPA1 16 kDa protein
62	8.124999702	IPI00644968,1	HNRPA1L3 Putative heterogeneous nuclear ribonucleoprotein A1-like protein 3
62	8.124999702	IPI00465365,4	HNRNPA1 Isoform A1-A of Heterogeneous nuclear ribonucleoprotein A1
62	6.989247352	IPI00215965,2	HNRNPA1 Isoform A1-B of Heterogeneous nuclear ribonucleoprotein A1
62	6.030150875	IPI00411329,4	- Putative uncharacterized protein ENSP00000222956 (Fragment)
62	4.494382069	IPI00879518,1	- 29 kDa protein
62	4.494382069	IPI00797148,1	HNRNPA1 Isoform 2 of Heterogeneous nuclear ribonucleoprotein A1
62	4.477611929	IPI00789127,1	- 30 kDa protein
62	4.270462692	IPI00176692,7	- 32 kDa protein
62	3.986711055	IPI00738822,1	LOC645691 similar to heterogeneous nuclear ribonucleoprotein A1
62	3.986711055	IPI00478539,2	LOC645691 similar to heterogeneous nuclear ribonucleoprotein A1
62	3.738317639	IPI00760620,2	HNRPA1L-2 Similar to Heterogeneous nuclear ribonucleoprotein A1
62	2.500000037	IPI00879501,2	HNRNPA1L2 Heterogeneous nuclear ribonucleoprotein A1-like protein
63	14.45783079	IPI00413344,3	CFL2 Cofilin-2

63	12.04819307	IPI00012011,6	CFL1 Cofilin-1
63	9.821428359	IPI00909841,1	- cDNA FLJ51435, moderately similar to Cofilin-1
64	4.031209275	IPI00291792,2	ITGB2 Integrin beta-2
64	1.825842634	IPI00103356,2	ITGB2 Integrin beta
65	6.425702572	IPI00386854,6	HNRNPA2B1 28 kDa protein
65	5.047318712	IPI00916517,1	HNRNPA2B1 34 kDa protein
65	4.692082107	IPI00414696,1	HNRNPA2B1 Isoform A2 of Heterogeneous nuclear ribonucleoproteins A2/B1
65	4.532577842	IPI00396378,3	HNRNPA2B1 Isoform B1 of Heterogeneous nuclear ribonucleoproteins A2/B1
65	3.864734247	IPI00874030,3	HNRNPA2B1 43 kDa protein
66	8.14479664	IPI00910724,1	- cDNA FLJ60170, highly similar to Guanine nucleotide-binding protein subunit beta 2-like 1
66	5.678233504	IPI00848226,1	GNB2L1 Guanine nucleotide-binding protein subunit beta-2-like 1
66	5.202312022	IPI00641950,4	GNB2L1 38 kDa protein
67	12.59259284	IPI00910666,1	- cDNA FLJ52993, highly similar to Heterogeneous nuclear ribonucleoprotein C
67	11.5646258	IPI00910718,1	- cDNA FLJ52975, highly similar to Heterogeneous nuclear ribonucleoproteins C
67	6.80000037	IPI00759596,1	HNRNPC Isoform 4 of Heterogeneous nuclear ribonucleoproteins C1/C2
67	5.902777612	IPI00909232,1	- cDNA FLJ53542, highly similar to Heterogeneous nuclear ribonucleoproteins C
67	5.802047625	IPI00216592,2	HNRNPC Isoform C1 of Heterogeneous nuclear ribonucleoproteins C1/C2
67	5.555555597	IPI00477313,3	HNRNPC Isoform C2 of Heterogeneous nuclear ribonucleoproteins C1/C2
68	8.994708955	IPI00892793,1	CTSD 20 kDa protein
68	4.126213491	IPI00011229,1	CTSD Cathepsin D
68	2.374670096	IPI00853455,1	CTSD Protein
69	4.166666791	IPI00872814,1	MSN Putative uncharacterized protein MSN (Fragment)
69	4.159445316	IPI00219365,3	MSN Moesin
70	2.564102598	IPI00872773,1	ERO1L Putative uncharacterized protein ERO1L
70	2.564102598	IPI00386755,2	ERO1L ERO1-like protein alpha
71	5.32544367	IPI00892511,1	PPP1CB 20 kDa protein
71	5.027933046	IPI00894274,2	PPP1CB cDNA FLJ58972, highly similar to Serine/threonine-protein phosphatase PP1-beta catalytic subunit
71	3.651685268	IPI00871289,1	PPP1CB 40 kDa protein
71	3.542234376	IPI00872177,1	PPP1CB 41 kDa protein
71	3.146853298	IPI00410128,2	PPP1CA protein phosphatase 1, catalytic subunit, alpha isoform 2
71	2.786377631	IPI00005705,1	PPP1CC Isoform Gamma-1 of Serine/threonine-protein phosphatase PP1-gamma catalytic subunit
71	2.752293646	IPI00218236,6	PPP1CB Serine/threonine-protein phosphatase PP1-beta catalytic subunit
71	2.727272734	IPI00550451,1	PPP1CA Serine/threonine-protein phosphatase PP1-alpha catalytic subunit

71	2.670623176	IPI00218187,1	PPP1CC Isoform Gamma-2 of Serine/threonine-protein phosphatase PP1-gamma catalytic subunit
71	2.639296278	IPI00027423,3	PPP1CA protein phosphatase 1, catalytic subunit, alpha isoform 3
71	2.179176733	IPI00902512,1	- cDNA FLJ45714 fis, clone FEKID2002637, highly similar to Serine/threonine-protein phosphatase PP1-alphacatalytic
72	10.78431383	IPI00796467,1	TF 11 kDa protein
72	1.575931162	IPI00022463,1	TF Serotransferrin
73	11.88811213	IPI00792951,1	CSRP2 16 kDa protein
73	8.808290213	IPI00002824,7	CSRP2 Cysteine and glycine-rich protein 2
74	7.017543912	IPI00887294,1	LOC100132353 similar to GDP dissociation inhibitor 2
74	3.999999911	IPI00640006,1	GDI2 GDP dissociation inhibitor 2 isoform 2
74	3.563474491	IPI00031461,2	GDI2 cDNA FLJ60299, highly similar to Rab GDP dissociation inhibitor beta
75	4.174572974	IPI00465436,4	CAT Catalase
76	3.791469336	IPI00465361,4	RPL13 60S ribosomal protein L13
77	12.62135953	IPI00453473,6	HIST1H4H;HIST2H4A;HIST4H4;HIST1H4F;HIST1H4D;HIST1H4K;HIST1H4C;HIST1H4J;HIST1H4A;HIST1H4I;HIST1H4B;HIST2H4B;HIST1H4E;HIST1H4L Histone H4
78	9.025270492	IPI00290462,5	CBR3 Carbonyl reductase [NADPH] 3
78	7.514450699	IPI00909888,1	- cDNA FLJ60474, highly similar to Carbonyl reductase
78	5.855855718	IPI00795334,1	CBR1 Putative uncharacterized protein CBR1
78	4.693140835	IPI00295386,7	CBR1 Carbonyl reductase [NADPH] 1
79	9.448818862	IPI00169276,2	PRSS1 Putative trypsin-6
79	5.263157934	IPI00843764,2	PRSS3 Isoform C of Trypsin-3
79	5.263157934	IPI00815665,1	PRSS1 PRSS1 protein
79	5.263157934	IPI00011694,1	PRSS1 Trypsin-1
79	5.000000075	IPI00220839,4	PRSS3 Isoform B of Trypsin-3
79	4.980842769	IPI00385250,1	PRSS3 Protease serine 4 isoform B
79	4.980842769	IPI00011695,8	PRSS2 Protease serine 2 isoform B
79	4.276315868	IPI00015614,4	PRSS3 Isoform A of Trypsin-3
80	4.950495064	IPI00794403,1	LUM 23 kDa protein
80	4.733727872	IPI00020986,2	LUM Lumican
80	4.310344905	IPI00796888,1	LUM 26 kDa protein
81	7.299269736	IPI00793806,1	PSMD2 15 kDa protein
81	1.982378773	IPI00012268,3	PSMD2 26S proteasome non-ATPase regulatory subunit 2
81	1.692047343	IPI00384420,1	PSMD2 P67
82	2.100840397	IPI00011285,1	CAPN1 Calpain-1 catalytic subunit

83	16.12903178	IPI00007047,1	S100A8 Protein S100-A8
84	11.23595536	IPI00917959,1	HLA-DRB5;ZNF749;LOC100133661;HLA-DRB4;LOC100133484;HLA-DRB2;HLA-DRB1;HLA-
84	11.23595536	IPI00917080,1	HLA-DRB5;ZNF749;LOC100133661;HLA-DRB4;LOC100133484;HLA-DRB2;HLA-DRB1;HLA-
84	11.23595536	IPI00914993,1	HLA-DRB5;ZNF749;LOC100133661;HLA-DRB4;LOC100133484;HLA-DRB2;HLA-DRB1;HLA-DRB3;LOC100133811;RNASE2 Leucocyte antigen DR beta 1 chain (Fragment)
84	9.900990129	IPI00915979,1	HLA-DRB5;ZNF749;LOC100133661;HLA-DRB4;LOC100133484;HLA-DRB2;HLA-DRB1;HLA-
84	3.759398311	IPI00746667,1	HLA-DRB5;ZNF749;LOC100133661;HLA-DRB4;LOC100133484;HLA-DRB2;HLA-DRB1;HLA-DRB3;LOC100133811;RNASE2 HLA class II histocompatibility antigen, DRB1-11 beta chain
84	3.759398311	IPI00005180,2	HLA-DRB5;ZNF749;LOC100133661;HLA-DRB4;LOC100133484;HLA-DRB2;HLA-DRB1;HLA-DRB3;LOC100133811;RNASE2 HLA class II histocompatibility antigen, DRB1-8 beta chain
85	8.870967478	IPI00513775,1	PHPT1 phosphohistidine phosphatase 1 isoform 2
85	8.799999952	IPI00914536,1	PHPT1 phosphohistidine phosphatase 1 isoform 1
85	8.799999952	IPI00299977,1	PHPT1 14 kDa phosphohistidine phosphatase
86	3.778337687	IPI00910487,1	- cDNA FLJ52569, highly similar to Collagen-binding protein 2
86	3.588516638	IPI00032140,4	SERPINH1 Serpin H1
87	10.29411778	IPI00784001,5	- Similar to His3
87	6.542056054	IPI00874018,1	LOC644914 Histone H3 (Fragment)
87	6.194690242	IPI00909530,1	- cDNA FLJ52843, highly similar to Histone H3,3
87	5.691057071	IPI00477080,1	- cDNA FLJ57905, moderately similar to Histone H3,3
87	5.147058889	IPI00465070,7	HIST1H3C;HIST1H3F;HIST1H3G;HIST1H3J;HIST1H3B;HIST1H3H;HIST1H3I;HIST1H3D;HIST1H3A;HIST1H3E;HIST1H2AD Histone H3,1
87	5.147058889	IPI00219038,9	H3F3B;LOC440926;H3F3A Histone H3,3
87	5.147058889	IPI00216402,3	HIST3H3 Histone H3,1t
87	5.147058889	IPI00171611,7	HIST2H3A;HIST2H3D;HIST2H3C Histone H3,2
88	10.00000015	IPI00909453,1	- cDNA FLJ52243, highly similar to Heat-shock protein beta-1
88	8.292683214	IPI00025512,2	HSPB1 Heat shock protein beta-1
89	2.985074557	IPI00909338,1	- cDNA FLJ51982, highly similar to Lysosome membrane protein 2
89	2.092050202	IPI00217766,3	SCARB2 Lysosome membrane protein 2
90	11.34020612	IPI00641244,1	PRDX1 11 kDa protein
90	6.432748586	IPI00640741,1	PRDX1 19 kDa protein
90	6.010929123	IPI00909207,1	- cDNA FLJ60461, highly similar to Peroxiredoxin-2
90	5.555555597	IPI00027350,3	PRDX2 Peroxiredoxin-2
90	5.527638271	IPI00000874,1	PRDX1 Peroxiredoxin-1

91	3.97727266	IPI00896370,2	SOD2 cDNA FLJ40076 fis, clone TESTI2000874, highly similar to Superoxide dismutase
91	3.825136647	IPI00607577,2	SOD2 manganese superoxide dismutase isoform B precursor
91	3.153153136	IPI00847322,1	SOD2 manganese superoxide dismutase isoform A precursor
91	3.153153136	IPI00022314,1	SOD2 Superoxide dismutase [Mn], mitochondrial
92	3.750000149	IPI00556640,1	PSAP PSAP protein
92	1.717557199	IPI00012503,1	PSAP Isoform Sap-mu-0 of Proactivator polypeptide
92	1.711026579	IPI00873201,1	PSAP Isoform Sap-mu-6 of Proactivator polypeptide
92	1.707779802	IPI00873020,1	PSAP Prosaposin variant
92	1.707779802	IPI00744835,1	PSAP Isoform Sap-mu-9 of Proactivator polypeptide
92	1.61001794	IPI00219825,2	PSAP Prosaposin
93	2.150537632	IPI00794875,1	PA2G4 41 kDa protein
93	2.030456811	IPI00299000,5	PA2G4 Proliferation-associated protein 2G4
93	1.970443316	IPI00807557,1	PA2G4 PA2G4 protein (Fragment)
94	5.35714291	IPI00220301,5	PRDX6 Peroxiredoxin-6
95	1.333333366	IPI00219516,2	GUSB Isoform Short of Beta-glucuronidase
95	1.228878647	IPI00027745,4	GUSB Isoform Long of Beta-glucuronidase
96	2.994011901	IPI00219217,3	LDHB L-lactate dehydrogenase B chain
97	9.090909362	IPI00215611,5	CRIP1 Cysteine-rich protein 1
98	3.587444127	IPI00022391,1	APCS Serum amyloid P-component
99	9.574468434	IPI00021827,3	DEFA3 Neutrophil defensin 3
99	9.574468434	IPI00005721,1	DEFA1;LOC728358 Neutrophil defensin 1
100	8.585858345	IPI00186793,6	PTK7 Putative uncharacterized protein DKFZp434L0319
100	4.787234217	IPI00168813,1	PTK7 PTK7 protein tyrosine kinase 7 isoform c precursor
100	4.578313231	IPI00903118,1	- cDNA FLJ31086 fis, clone IMR321000044, highly similar to Tyrosine-protein kinase-like 7
100	4.437869787	IPI00478565,2	PTK7 PTK7 protein tyrosine kinase 7 isoform d precursor
100	4.368932173	IPI00170814,1	PTK7 PTK7 protein tyrosine kinase 7 isoform b precursor
100	4.205607623	IPI00298292,2	PTK7 Tyrosine-protein kinase-like 7
101	5.66666685	IPI00005161,3	ARPC2 Actin-related protein 2/3 complex subunit 2
102	2.826855145	IPI00909956,1	- cDNA FLJ59103, highly similar to T-complex protein 1 subunit epsilon
102	1.556420233	IPI00910316,1	- cDNA FLJ54333, highly similar to T-complex protein 1 subunit epsilon
102	1.478743088	IPI00010720,1	CCT5 T-complex protein 1 subunit epsilon
103	1.50214592	IPI00254338,2	FAM134C Protein FAM134C
104	29.10447717	IPI00477513,2	- Putative uncharacterized protein ENSP00000346992 (Fragment)

104	27.46478915	IPI00217473,5	HBZ Hemoglobin subunit zeta
104	15.49295783	IPI00853068,1	HBA1;HBA2 Alpha 2 globin variant (Fragment)
104	15.49295783	IPI00410714,5	HBA1;HBA2 Hemoglobin subunit alpha
105	7.377049327	IPI00759832,1	YWHAB Isoform Short of 14-3-3 protein beta/alpha
105	7.317072898	IPI00216318,5	YWHAB Isoform Long of 14-3-3 protein beta/alpha
106	2.085620165	IPI00013808,1	ACTN4 Alpha-actinin-4
106	1.923076995	IPI00908776,1	- cDNA FLJ61380, highly similar to Alpha-actinin-4
106	1.423487533	IPI00908458,1	- cDNA FLJ58087, highly similar to Alpha-actinin-4
107	10.48951074	IPI00216952,1	LMNA Isoform C of Lamin-A/C
107	9.771987051	IPI00644087,1	LMNA Lamin A/C
107	9.463722259	IPI00216953,1	LMNA Isoform ADelta10 of Lamin-A/C
107	9.036144614	IPI00021405,3	LMNA Isoform A of Lamin-A/C
107	8.961303532	IPI00514817,5	LMNA Lamin A/C
107	5.653021485	IPI00514320,3	LMNA Lamin A/C
107	5.05226478	IPI00910241,1	- cDNA FLJ56081, highly similar to Lamin-A/C
108	4.45269011	IPI00604607,2	HSP90AA1 Hsp89-alpha-delta-N
108	4.234972596	IPI00784295,2	HSP90AA1 Isoform 1 of Heat shock protein HSP 90-alpha
108	3.629976511	IPI00382470,3	HSP90AA1 heat shock protein 90kDa alpha (cytosolic), class A member 1 isoform 1
109	8.979591727	IPI00853525,1	APOA1 Apolipoprotein A1
109	8.239700645	IPI00021841,1	APOA1 Apolipoprotein A-I

Enriched tumor cell suspension

**N**	**%Cov**	**Accession**	**Name**
1	8.331491798	IPI00420096,4	PLEC1 plectin 1 isoform 3
1	8.2275711	IPI00398002,6	PLEC1 Isoform 3 of Plectin-1
1	8.062015474	IPI00398776,3	PLEC1 plectin 1 isoform 7
1	8.030002564	IPI00398775,3	PLEC1 plectin 1 isoform 2
1	8.005278558	IPI00398779,5	PLEC1 Isoform 4 of Plectin-1
1	8.005278558	IPI00398777,3	PLEC1 plectin 1 isoform 8
1	7.998242229	IPI00398778,3	PLEC1 plectin 1 isoform 10
1	7.95802325	IPI00186711,4	PLEC1 Isoform 2 of Plectin-1
1	7.899231464	IPI00014898,3	PLEC1 Isoform 1 of Plectin-1
2	29.51289415	IPI00747707,2	KRT17 Radiated keratinocyte mRNA 266

2	28.0092597	IPI00450768,7	KRT17 Keratin, type I cytoskeletal 17
3	29.1958034	IPI00216952,1	LMNA Isoform C of Lamin-A/C
3	27.19869614	IPI00644087,1	LMNA Lamin A/C
3	25.15060306	IPI00021405,3	LMNA Isoform A of Lamin-A/C
3	24.13249165	IPI00216953,1	LMNA Isoform ADelta10 of Lamin-A/C
4	19.0476194	IPI00796776,1	KRT5 cDNA FLJ54081, highly similar to Keratin, type II cytoskeletal 5
4	18.30508411	IPI00009867,3	KRT5 Keratin, type II cytoskeletal 5
5	42.73504317	IPI00894365,2	ACTB cDNA FLJ52842, highly similar to Actin, cytoplasmic 1
5	40.0000006	IPI00894498,1	ACTB Beta actin variant (Fragment)
5	40.0000006	IPI00021440,1	ACTG1 Actin, cytoplasmic 2
5	40.0000006	IPI00021439,1	ACTB Actin, cytoplasmic 1
6	8.724489808	IPI00019502,3	MYH9 Isoform 1 of Myosin-9
7	32.84457624	IPI00414696,1	HNRNPA2B1 Isoform A2 of Heterogeneous nuclear ribonucleoproteins A2/B1
7	31.72804415	IPI00396378,3	HNRNPA2B1 Isoform B1 of Heterogeneous nuclear ribonucleoproteins A2/B1
7	27.05313861	IPI00874030,3	HNRNPA2B1 43 kDa protein
7	26.49842203	IPI00916517,1	HNRNPA2B1 34 kDa protein
7	25.70281029	IPI00386854,6	HNRNPA2B1 28 kDa protein
8	19.49999928	IPI00879819,1	LMNB2 Lamin-B2
8	18.87096763	IPI00009771,6	LMNB2 Lamin-B2
9	44.99999881	IPI00479145,2	KRT19 Keratin, type I cytoskeletal 19
10	30.84886074	IPI00554648,3	KRT8 Keratin, type II cytoskeletal 8
11	27.90697813	IPI00790831,1	LMNB1 LMNB1 protein
11	21.16040885	IPI00217975,4	LMNB1 Lamin-B1
12	10.21650881	IPI00029818,5	MYH14 Isoform 5 of Myosin-14
12	9.885172546	IPI00337335,6	MYH14 Isoform 1 of Myosin-14
12	9.72495079	IPI00607818,2	MYH14 MYH14 variant protein
12	9.162450582	IPI00607778,1	MYH14 Isoform 4 of Myosin-14
13	18.22541952	IPI00384697,2	ALB Isoform 2 of Serum albumin
13	17.20647812	IPI00878517,1	ALB 56 kDa protein
13	16.25238955	IPI00908876,1	- cDNA FLJ50830, highly similar to Serum albumin
13	13.95730674	IPI00745872,2	ALB Isoform 1 of Serum albumin
13	13.55661899	IPI00022434,4	ALB Putative uncharacterized protein ALB
14	21.90476209	IPI00873598,1	KRT13 46 kDa protein

14	21.90476209	IPI00171196,2	KRT13 keratin 13 isoform b
14	20.087336	IPI00009866,6	KRT13 Isoform 1 of Keratin, type I cytoskeletal 13
14	19.71831024	IPI00550661,2	KRT13 Isoform 2 of Keratin, type I cytoskeletal 13
15	20.60085833	IPI00418471,6	VIM Vimentin
16	8.011049777	IPI00414676,6	HSP90AB1 Heat shock protein HSP 90-beta
17	22.3140493	IPI00909140,1	- cDNA FLJ53012, highly similar to Tubulin beta-7 chain
17	15.0234744	IPI00645452,1	TUBB Tubulin, beta polypeptide
17	14.83146101	IPI00013475,1	TUBB2A Tubulin beta-2A chain
17	14.41441476	IPI00011654,2	TUBB Tubulin beta chain
17	13.9784947	IPI00647896,1	TUBB Tubulin, beta
17	11.68539301	IPI00031370,3	TUBB2B Tubulin beta-2B chain
17	10.72555184	IPI00908770,1	- cDNA FLJ53063, highly similar to Tubulin beta-7 chain
17	10.58201045	IPI00640115,1	TUBB3 HCG1983504, isoform CRA_f
17	8.948545903	IPI00646779,2	TUBB6 TUBB6 protein
17	8.888889104	IPI00013683,2	TUBB3 Tubulin beta-3 chain
17	5.018820614	IPI00152453,1	TUBB3 HCG2042771
18	14.45427686	IPI00455315,4	ANXA2 Annexin A2
18	14.45427686	IPI00334627,3	ANXA2P2 Putative annexin A2-like protein
18	13.72549087	IPI00418169,3	ANXA2 annexin A2 isoform 1
19	23.9700377	IPI00021841,1	APOA1 Apolipoprotein A-I
20	7.670043409	IPI00383296,5	HNRNPM Isoform 2 of Heterogeneous nuclear ribonucleoprotein M
20	7.260274142	IPI00171903,2	HNRNPM Isoform 1 of Heterogeneous nuclear ribonucleoprotein M
21	16.92650318	IPI00013881,6	HNRNPH1 Heterogeneous nuclear ribonucleoprotein H
21	16.1016956	IPI00479191,2	HNRNPH1 51 kDa protein
22	21.72284573	IPI00879518,1	- 29 kDa protein
22	21.72284573	IPI00797148,1	HNRNPA1 Isoform 2 of Heterogeneous nuclear ribonucleoprotein A1
22	18.1250006	IPI00879501,2	HNRNPA1L2 Heterogeneous nuclear ribonucleoprotein A1-like protein
22	18.1250006	IPI00644968,1	HNRPA1L3 Putative heterogeneous nuclear ribonucleoprotein A1-like protein 3
22	18.1250006	IPI00465365,4	HNRNPA1 Isoform A1-A of Heterogeneous nuclear ribonucleoprotein A1
22	18.06853563	IPI00760620,2	HNRPA1L-2 Similar to Heterogeneous nuclear ribonucleoprotein A1
22	15.59139788	IPI00215965,2	HNRNPA1 Isoform A1-B of Heterogeneous nuclear ribonucleoprotein A1
23	10.75268835	IPI00027146,1	GLUD2 Glutamate dehydrogenase 2, mitochondrial
23	10.75268835	IPI00016801,1	GLUD1 Glutamate dehydrogenase 1, mitochondrial

24	17.75700897	IPI00646877,7	SFTPA2;SFTPA2B;SFTPA1B;SFTPA1 cDNA FLJ54288, moderately similar to Pulmonary surfactant-associated protein A1
24	16.45021588	IPI00739950,8	SFTPA1B;SFTPA1 cDNA FLJ51913, highly similar to Pulmonary surfactant-associated protein A1
24	15.32258093	IPI00293120,5	SFTPA2;SFTPA2B Pulmonary surfactant-associated protein A2
24	15.32258093	IPI00012889,2	SFTPA1B;SFTPA1 Pulmonary surfactant-associated protein A1
25	17.10526347	IPI00745267,2	TPM1 Isoform 2 of Tropomyosin alpha-1 chain
25	16.04938209	IPI00018853,1	TPM1 Tropomyosin isoform
25	15.72580636	IPI00384369,4	TPM1 Tropomyosin 1 alpha variant 6
25	15.72580636	IPI00216134,3	TPM1 tropomyosin 1 alpha chain isoform 7
25	13.73239458	IPI00296039,7	TPM1 Isoform 4 of Tropomyosin alpha-1 chain
25	13.73239458	IPI00216135,1	TPM1 Isoform 3 of Tropomyosin alpha-1 chain
25	12.32394353	IPI00915324,1	TPM1 Isoform 6 of Tropomyosin alpha-1 chain
25	12.32394353	IPI00604537,2	TPM1 tropomyosin 1 alpha chain isoform 3
25	12.32394353	IPI00014581,1	TPM1 Isoform 1 of Tropomyosin alpha-1 chain
25	12.32394353	IPI00000230,6	TPM1 tropomyosin 1 alpha chain isoform 2
26	34.73053873	IPI00471928,5	ATP5A1 cDNA FLJ54625, highly similar to ATP synthase subunit alpha, mitochondrial
26	33.5260123	IPI00641249,2	ATP5A1 18 kDa protein
26	26.72811151	IPI00549805,3	ATP5A1 23 kDa protein
26	14.28571492	IPI00440493,2	ATP5A1 ATP synthase subunit alpha, mitochondrial
27	12.59079874	IPI00908762,1	- cDNA FLJ53509, highly similar to Galectin-3-binding protein
27	10.72164923	IPI00902654,2	- cDNA FLJ54583, highly similar to Galectin-3-binding protein
27	9.386281669	IPI00887555,1	LOC100133842 similar to lectin, galactoside-binding, soluble, 3 binding protein
27	8.888889104	IPI00023673,1	LGALS3BP Galectin-3-binding protein
28	29.10447717	IPI00794807,1	KRT18 15 kDa protein
28	17.90697724	IPI00554788,5	KRT18 Keratin, type I cytoskeletal 18
28	6.88912794	IPI00888063,1	KRT18P33 similar to hCG21219
29	4.190389439	IPI00024284,5	HSPG2 Basement membrane-specific heparan sulfate proteoglycan core protein
30	13.19942623	IPI00903112,1	LTF cDNA FLJ36533 fis, clone TRACH2004428, highly similar to Lactotransferrin (Fragment)
30	11.56156138	IPI00789477,1	LTF Truncated lactoferrin
30	11.53305173	IPI00298860,5	LTF Growth-inhibiting protein 12
30	11.1328125	IPI00909837,1	LOC728320 hypothetical protein
31	7.942973822	IPI00298497,3	FGB Fibrinogen beta chain
32	14.81481493	IPI00910666,1	- cDNA FLJ52993, highly similar to Heterogeneous nuclear ribonucleoprotein C

32	13.60544264	IPI00910718,1	- cDNA FLJ52975, highly similar to Heterogeneous nuclear ribonucleoproteins C
32	11.99999973	IPI00759596,1	HNRNPC Isoform 4 of Heterogeneous nuclear ribonucleoproteins C1/C2
32	10.41666642	IPI00909232,1	- cDNA FLJ53542, highly similar to Heterogeneous nuclear ribonucleoproteins C
32	10.23890749	IPI00216592,2	HNRNPC Isoform C1 of Heterogeneous nuclear ribonucleoproteins C1/C2
32	9.803921729	IPI00477313,3	HNRNPC Isoform C2 of Heterogeneous nuclear ribonucleoproteins C1/C2
32	6.48464188	IPI00887991,1	LOC649330 similar to heterogeneous nuclear ribonucleoprotein C-like 1
32	6.48464188	IPI00735540,1	LOC440563 LOC440563 protein
32	6.48464188	IPI00027569,1	HNRNPCL1 Heterogeneous nuclear ribonucleoprotein C-like 1
32	6.375838816	IPI00887544,1	LOC652665 similar to heterogeneous nuclear ribonucleoprotein C-like 1, partial
33	27.41935551	IPI00843996,1	SFRS3 cDNA FLJ52832, highly similar to Splicing factor, arginine/serine-rich 3
33	20.73170692	IPI00010204,1	SFRS3 Splicing factor, arginine/serine-rich 3
34	6.751054525	IPI00742696,2	GC vitamin D-binding protein precursor
34	6.751054525	IPI00555812,4	GC Vitamin D-binding protein
35	11.20797023	IPI00027230,3	HSP90B1 Endoplasmin
35	10.50724611	IPI00908897,1	- cDNA FLJ58626, highly similar to Endoplasmin
36	18.2432428	IPI00019038,1	LYZ Lysozyme C
37	5.39881587	IPI00013933,2	DSP Isoform DPI of Desmoplakin
38	28.07017565	IPI00027462,1	S100A9 Protein S100-A9
39	8.943089098	IPI00744148,2	H2AFY Isoform 1 of Core histone macro-H2A,1
39	8.894878626	IPI00059366,4	H2AFY H2A histone family, member Y isoform 2
39	8.870967478	IPI00304171,6	H2AFY Isoform 2 of Core histone macro-H2A,1
39	8.854166418	IPI00148096,2	H2AFY H2A histone family, member Y isoform 2 variant (Fragment)
39	8.500000089	IPI00910098,1	- cDNA FLJ55076, highly similar to Core histone macro-H2A,1
40	14.67304677	IPI00010471,5	LCP1 Plastin-2
41	47.55244851	IPI00789823,1	UBB;RPS27A;UBC 16 kDa protein
41	46.7889905	IPI00790633,1	UBB;RPS27A;UBC 25 kDa protein
41	45.86894512	IPI00792712,1	UBB;RPS27A;UBC 39 kDa protein
41	45.40540576	IPI00795527,1	UBB;RPS27A;UBC 21 kDa protein
41	44.67153251	IPI00798127,1	UBB;RPS27A;UBC ubiquitin C
41	44.59016323	IPI00793729,1	UBB;RPS27A;UBC UBC protein
41	44.54148412	IPI00719280,2	UBB;RPS27A;UBC ubiquitin B precursor
41	44.44444478	IPI00796007,2	UBB;RPS27A;UBC cDNA FLJ51326, highly similar to Homo sapiens ubiquitin B (UBB), mRNA
41	44.44444478	IPI00794925,1	UBB;RPS27A;UBC 21 kDa protein

41	43.58974397	IPI00794211,1	UBB;RPS27A;UBC 18 kDa protein
41	43.31210256	IPI00796600,1	UBB;RPS27A;UBC 18 kDa protein
41	42.01031029	IPI00744274,1	UBB;RPS27A;UBC 44 kDa protein
41	42.01031029	IPI00743650,1	UBB;RPS27A;UBC 44 kDa protein
41	42.01031029	IPI00743241,1	UBB;RPS27A;UBC 44 kDa protein
41	41.88679159	IPI00789107,1	UBB;RPS27A;UBC 30 kDa protein
41	41.46341383	IPI00792139,1	UBB;RPS27A;UBC Ubiquitin C
41	40.71856141	IPI00797400,1	UBB;RPS27A;UBC 19 kDa protein
41	40.0000006	IPI00793810,1	UBB;RPS27A;UBC 19 kDa protein
41	38.56209219	IPI00784990,2	UBB;RPS27A;UBC Ubiquitin C splice variant
41	35.78947484	IPI00654754,1	UBB;RPS27A;UBC RPS27A protein
41	32.07547069	IPI00798155,4	UBB;RPS27A;UBC Ubiquitin C splice variant
41	31.19266033	IPI00793330,1	UBB;RPS27A;UBC 12 kDa protein
41	29.69432175	IPI00794205,1	- 26 kDa protein
41	26.5625	IPI00456429,3	UBA52;UBB;RPS27A;UBC ubiquitin and ribosomal protein L40 precursor
41	26.33928657	IPI00418813,2	- Similar to Ribosomal protein S27a
41	21.79487199	IPI00179330,6	UBB;RPS27A;UBC ubiquitin and ribosomal protein S27a precursor
41	14.10256475	IPI00397808,3	LOC388720 similar to ubiquitin
42	6.321370602	IPI00843765,1	SPTAN1 Isoform 3 of Spectrin alpha chain, brain
42	6.321370602	IPI00744706,2	SPTAN1 cDNA FLJ61399, highly similar to Spectrin alpha chain, brain
42	6.270226836	IPI00844215,1	SPTAN1 Isoform 1 of Spectrin alpha chain, brain
42	6.25756979	IPI00879810,1	SPTAN1 Putative uncharacterized protein SPTAN1
42	6.25756979	IPI00871535,1	SPTAN1 Isoform 2 of Spectrin alpha chain, brain
43	28.78504694	IPI00909059,1	- cDNA FLJ53910, highly similar to Keratin, type II cytoskeletal 6A
43	27.30496526	IPI00300725,7	KRT6A Keratin, type II cytoskeletal 6A
43	24.82269555	IPI00293665,8	KRT6B Keratin, type II cytoskeletal 6B
43	24.46808517	IPI00299145,9	KRT6C Keratin, type II cytoskeletal 6C
43	20.15209198	IPI00910738,1	- cDNA FLJ60647, highly similar to Keratin, type II cytoskeletal 6B
44	6.663493812	IPI00006196,3	NUMA1 Isoform 2 of Nuclear mitotic apparatus protein 1
44	6.61938563	IPI00872028,1	NUMA1 NUMA1 variant protein
44	6.61938563	IPI00292771,4	NUMA1 Isoform 1 of Nuclear mitotic apparatus protein 1
45	11.60409525	IPI00795257,1	GAPDH 32 kDa protein
45	10.1492539	IPI00219018,7	GAPDH Glyceraldehyde-3-phosphate dehydrogenase

46	8.153477311	IPI00909073,1	- cDNA FLJ53752, highly similar to Heat shock 70 kDa protein 1
46	4.411764815	IPI00647012,2	HSPA1A;HSPA1B cDNA FLJ54389, highly similar to Heat shock 70 kDa protein 1
46	4.262877628	IPI00910482,1	- cDNA FLJ54407, highly similar to Heat shock 70 kDa protein 1
46	4.095563293	IPI00911039,1	- cDNA FLJ54408, highly similar to Heat shock 70 kDa protein 1
46	3.744149581	IPI00845339,1	HSPA1A;HSPA1B cDNA FLJ54392, highly similar to Heat shock 70 kDa protein 1
46	3.744149581	IPI00304925,5	HSPA1A;HSPA1B Heat shock 70 kDa protein 1
47	3.721781448	IPI00455383,4	CLTC Isoform 2 of Clathrin heavy chain 1
47	3.641790897	IPI00024067,4	CLTC Isoform 1 of Clathrin heavy chain 1
48	20.89552283	IPI00787441,2	- 7 kDa protein
48	19.0476194	IPI00816252,1	H2BFS Histone H2B
48	19.0476194	IPI00815755,1	HIST1H2BI;HIST1H2BE;HIST1H2BG;HIST1H2BF;HIST1H2BC Histone H2B
48	19.0476194	IPI00554798,2	HIST1H2BM Histone H2B type 1-M
48	19.0476194	IPI00515061,3	HIST1H2BJ Histone H2B type 1-J
48	19.0476194	IPI00477495,3	H2BFS Histone H2B type F-S
48	19.0476194	IPI00329665,8	HIST2H2BF;HIST2H2BA Histone H2B type 2-F
48	19.0476194	IPI00303133,8	HIST1H2BH Histone H2B type 1-H
48	19.0476194	IPI00220403,3	HIST1H2BB Histone H2B type 1-B
48	19.0476194	IPI00166293,5	HIST3H2BB Histone H2B type 3-B
48	19.0476194	IPI00152906,6	HIST1H2BD Histone H2B type 1-D
48	19.0476194	IPI00152785,3	HIST1H2BO Histone H2B type 1-O
48	19.0476194	IPI00020101,9	HIST1H2BI;HIST1H2BE;HIST1H2BG;HIST1H2BF;HIST1H2BC histone cluster 1, H2bg
48	19.0476194	IPI00018534,4	HIST1H2BL Histone H2B type 1-L
48	19.0476194	IPI00003935,6	HIST2H2BE Histone H2B type 2-E
48	17.91044772	IPI00419833,8	HIST1H2BK cDNA FLJ56780, highly similar to Histone H2B type 2-F
48	14.45783079	IPI00794461,1	HIST1H2BN Histone H2B type 1-N
48	14.45783079	IPI00646240,3	HIST2H2BF;HIST2H2BA cDNA FLJ56787, highly similar to Histone H2B type 2-F
48	11.02362201	IPI00465363,3	HIST1H2BA Histone H2B type 1-A
49	8.232711256	IPI00013808,1	ACTN4 Alpha-actinin-4
50	46.66666687	IPI00789536,1	- cDNA FLJ56791, highly similar to Keratin, type I cytoskeletal 16
50	34.28571522	IPI00795719,1	- cDNA FLJ53570, highly similar to Keratin, type I cytoskeletal 16
50	20.29598355	IPI00217963,3	KRT16 Keratin, type I cytoskeletal 16
50	18.00847501	IPI00384444,5	KRT14 Keratin, type I cytoskeletal 14
51	14.34426159	IPI00045498,4	HNRPDL Isoform 3 of Heterogeneous nuclear ribonucleoprotein D-like

51	11.62790731	IPI00845282,1	HNRPDL Isoform 2 of Heterogeneous nuclear ribonucleoprotein D-like
51	8.333333582	IPI00011274,3	HNRPDL Isoform 1 of Heterogeneous nuclear ribonucleoprotein D-like
52	6.590908766	IPI00644989,2	PDIA6 Isoform 1 of Protein disulfide-isomerase A6
52	5.89430891	IPI00299571,5	PDIA6 Isoform 2 of Protein disulfide-isomerase A6
53	10.56105644	IPI00026185,6	CAPZB Isoform 1 of F-actin-capping protein subunit beta
53	6.985294074	IPI00642256,2	CAPZB Isoform 2 of F-actin-capping protein subunit beta
53	6.375838816	IPI00641107,2	CAPZB cDNA, FLJ93598, highly similar to Homo sapiens capping protein (actin filament) muscle Z-line, beta (CAPZB), mRNA
53	5.671641976	IPI00218782,3	CAPZB cDNA FLJ60094, highly similar to F-actin capping protein subunit beta
54	27.52808928	IPI00873994,1	KRT15 Putative uncharacterized protein KRT15 (Fragment)
54	21.91235125	IPI00788699,2	KRT15 cDNA FLJ33920 fis, clone CTONG2016904, highly similar to KERATIN, TYPE I CYTOSKELETAL 15
54	21.05263174	IPI00290077,2	KRT15 Keratin, type I cytoskeletal 15
54	18.90034378	IPI00797326,1	KRT15 Putative uncharacterized protein KRT15
55	23.79032224	IPI00218820,1	TPM2 Isoform 3 of Tropomyosin beta chain
55	22.8873238	IPI00220709,3	TPM2 Isoform 2 of Tropomyosin beta chain
55	19.35483813	IPI00010779,4	TPM4 Isoform 1 of Tropomyosin alpha-4 chain
55	18.66197139	IPI00646748,1	TPM2 Tropomyosin 2
55	18.66197139	IPI00013991,1	TPM2 Isoform 1 of Tropomyosin beta chain
55	17.49174893	IPI00910712,1	- cDNA FLJ57036, highly similar to Homo sapiens tropomyosin 2 (beta) (TPM2), transcript variant 2, mRNA
55	16.45962745	IPI00513698,1	TPM2 Tropomyosin 2
55	12.32394353	IPI00216975,1	TPM4 Isoform 2 of Tropomyosin alpha-4 chain
56	6.275303662	IPI00397526,3	MYH10 Isoform 1 of Myosin-10
56	6.224899739	IPI00479307,4	MYH10 Isoform 2 of Myosin-10
56	6.209313869	IPI00790503,3	MYH10 Isoform 3 of Myosin-10
56	5.211558193	IPI00024870,1	MYH11 smooth muscle myosin heavy chain 11 isoform SM2A
56	5.192802101	IPI00744256,1	MYH11 smooth muscle myosin heavy chain 11 isoform SM2B
56	5.121703818	IPI00020501,1	MYH11 Myosin-11
56	5.103587732	IPI00873792,1	MYH11 228 kDa protein
56	5.103587732	IPI00743857,1	MYH11 smooth muscle myosin heavy chain 11 isoform SM1B
57	19.25465912	IPI00791554,1	KRT7 17 kDa protein
57	13.00639659	IPI00847342,1	KRT7 keratin 7
57	13.00639659	IPI00306959,10	KRT7 Keratin, type II cytoskeletal 7
58	4.680038244	IPI00026970,4	SUPT16H FACT complex subunit SPT16

59	2.896801382	IPI00009342,1	IQGAP1 Ras GTPase-activating-like protein IQGAP1
60	5.286839232	IPI00910438,1	SND1 cDNA FLJ54574, highly similar to Staphylococcal nuclease domain-containing protein 1
60	5.16483523	IPI00140420,4	SND1 Staphylococcal nuclease domain-containing protein 1
61	10.97178683	IPI00872379,1	ANXA5 Putative uncharacterized protein ANXA5 (Fragment)
61	10.9375	IPI00329801,12	ANXA5 Annexin A5
62	12.13017777	IPI00291006,2	MDH2 Malate dehydrogenase, mitochondrial
62	8.783783764	IPI00915384,1	- cDNA FLJ52880, highly similar to Malate dehydrogenase, mitochondrial
63	5.58035709	IPI00908449,1	- cDNA FLJ58737, highly similar to Splicing factor 3A subunit 3
63	4.990020022	IPI00029764,1	SF3A3 Splicing factor 3A subunit 3
64	10.44776142	IPI00884926,1	ORM1 orosomucoid 1 precursor
64	10.44776142	IPI00022429,3	ORM1 Alpha-1-acid glycoprotein 1
65	10.6250003	IPI00645745,1	G6PD 37 kDa protein
65	8.984375	IPI00642620,3	G6PD Glucose-6-phosphate dehydrogenase
65	6.862745434	IPI00455983,1	MAD2L1BP MAD2L1 binding protein isoform 1
65	6.692913175	IPI00884082,2	G6PD cDNA FLJ56794, highly similar to Glucose-6-phosphate 1-dehydrogenase
65	6.601941586	IPI00289800,7	G6PD Isoform Short of Glucose-6-phosphate 1-dehydrogenase
65	6.238532066	IPI00853547,1	G6PD glucose-6-phosphate dehydrogenase isoform a
65	6.060606241	IPI00216008,4	G6PD Isoform Long of Glucose-6-phosphate 1-dehydrogenase
65	2.389078587	IPI00014173,2	MAD2L1BP cDNA FLJ56354, highly similar to MAD2L1-binding protein
66	3.894080967	IPI00411462,7	FN1 Isoform 2 of Fibronectin
66	1.792828739	IPI00556632,3	FN1 Isoform 12 of Fibronectin
66	1.772525907	IPI00339224,1	FN1 Isoform 4 of Fibronectin
66	1.654411852	IPI00479723,3	FN1 Isoform 10 of Fibronectin
66	1.648351736	IPI00339226,1	FN1 Isoform 6 of Fibronectin
66	1.628222503	IPI00339225,1	FN1 Isoform 5 of Fibronectin
66	1.589404047	IPI00855777,1	FN1 Isoform 14 of Fibronectin
66	1.588001847	IPI00867588,1	FN1 Isoform 13 of Fibronectin
66	1.567944326	IPI00339228,1	FN1 Isoform 8 of Fibronectin
66	1.545064338	IPI00414283,5	FN1 fibronectin 1 isoform 4 preproprotein
66	1.528662443	IPI00339223,1	FN1 Isoform 3 of Fibronectin
66	1.508801337	IPI00339319,1	FN1 Isoform 11 of Fibronectin
66	1.508801337	IPI00022418,1	FN1 Isoform 1 of Fibronectin
66	1.508169249	IPI00873210,1	FN1 263 kDa protein

66	1.486988831	IPI00845263,1	FN1 fibronectin 1 isoform 2 preproprotein
66	1.471790671	IPI00339227,4	FN1 Isoform 7 of Fibronectin
66	1.453370973	IPI00855785,1	FN1 Isoform 15 of Fibronectin
67	9.375	IPI00879131,1	- 11 kDa protein
67	7.999999821	IPI00555602,2	EIF4A1 cDNA FLJ58012, moderately similar to Eukaryotic initiation factor 4A-I
67	6.650245935	IPI00871852,1	EIF4A1 46 kDa protein
67	6.650245935	IPI00025491,1	EIF4A1 Eukaryotic initiation factor 4A-I
67	4.136253148	IPI00009328,4	EIF4A3 Eukaryotic initiation factor 4A-III
67	3.196347132	IPI00788730,1	EIF4A2 25 kDa protein
67	2.800000086	IPI00794607,2	EIF4A2 cDNA FLJ58834, highly similar to Eukaryotic initiation factor 4A-II
67	2.243589796	IPI00030296,6	EIF4A2 Eukaryotic translation initiation factor 4A, isoform 2, isoform CRA_b
67	1.719901711	IPI00328328,3	EIF4A2 Isoform 1 of Eukaryotic initiation factor 4A-II
67	1.715686359	IPI00409717,1	EIF4A2 Isoform 2 of Eukaryotic initiation factor 4A-II
68	9.698996693	IPI00027252,6	PHB2 Prohibitin-2
68	9.578543901	IPI00797822,2	PHB2 cDNA FLJ56579, highly similar to Prohibitin-2
69	13.46153915	IPI00910830,1	- cDNA FLJ57715, highly similar to Voltage-dependent anion-selective channel protein 1
69	8.366534114	IPI00847300,1	- Similar to Voltage-dependent anion-selective channel protein 1
69	7.420494407	IPI00216308,5	VDAC1 Voltage-dependent anion-selective channel protein 1
70	7.046979666	IPI00155466,4	SUCLG2 Putative uncharacterized protein DKFZp586M2023
70	4.861111194	IPI00096066,2	SUCLG2 Succinyl-CoA ligase [GDP-forming] subunit beta, mitochondrial
70	2.156862803	IPI00164300,8	LOC283398 similar to hCG1791842 isoform 1
70	2.131783031	IPI00888064,1	LOC283398 similar to hCG1791842
71	8.231707662	IPI00011416,2	ECH1 Delta(3,5)-Delta(2,4)-dienoyl-CoA isomerase, mitochondrial
72	10.23622081	IPI00010796,1	P4HB Protein disulfide-isomerase
72	9.29203555	IPI00878551,2	P4HB cDNA FLJ59430, highly similar to Protein disulfide-isomerase
72	8.968609571	IPI00911004,1	- cDNA FLJ59939, highly similar to Protein disulfide-isomerase
73	7.153965533	IPI00339269,1	HSPA6 Heat shock 70 kDa protein 6
73	6.072874367	IPI00037070,3	HSPA8 Isoform 2 of Heat shock cognate 71 kDa protein
73	6.053268909	IPI00902596,1	- cDNA FLJ40505 fis, clone TESTI2045562, highly similar to HEAT SHOCK-RELATED 70 kDa PROTEIN 2
73	4.643962905	IPI00003865,1	HSPA8 Isoform 1 of Heat shock cognate 71 kDa protein
73	4.538341239	IPI00007702,1	HSPA2 Heat shock-related 70 kDa protein 2
74	23.79032224	IPI00215884,4	SFRS1 Isoform ASF-1 of Splicing factor, arginine/serine-rich 1
74	15.42288512	IPI00218592,5	SFRS1 Isoform ASF-3 of Splicing factor, arginine/serine-rich 1

74	10.61643809	IPI00218591,2	SFRS1 Isoform ASF-2 of Splicing factor, arginine/serine-rich 1
75	15.23809582	IPI00014055,3	NAPSA Napsin-A
75	9.662920982	IPI00793318,1	NAPSB Aspartyl protease 3
75	9.641255438	IPI00005800,2	- 48 kDa protein
76	5.514705926	IPI00798375,2	DDX5 cDNA FLJ59357, highly similar to Probable ATP-dependent RNA helicase DDX5
76	4.885993525	IPI00017617,1	DDX5 Probable ATP-dependent RNA helicase DDX5
77	13.45029175	IPI00640741,1	PRDX1 19 kDa protein
77	12.56830543	IPI00909207,1	- cDNA FLJ60461, highly similar to Peroxiredoxin-2
77	11.55778915	IPI00000874,1	PRDX1 Peroxiredoxin-1
77	11.34020612	IPI00641244,1	PRDX1 11 kDa protein
77	5.555555597	IPI00027350,3	PRDX2 Peroxiredoxin-2
78	6.15384616	IPI00009896,1	EPHX1 Epoxide hydrolase 1
79	3.949224204	IPI00005154,1	SSRP1 FACT complex subunit SSRP1
80	6.734006852	IPI00911034,1	- cDNA FLJ59360, moderately similar to Biglycan
80	3.583062068	IPI00643384,2	BGN Putative uncharacterized protein BGN
80	3.384615481	IPI00385748,1	BGN cDNA FLJ35635 fis, clone SPLEN2011805, highly similar to BONE/CARTILAGE PROTEOGLYCAN I
80	3.197674453	IPI00903084,1	- cDNA FLJ35704 fis, clone SPLEN2020183, highly similar to Biglycan
80	2.989130467	IPI00010790,1	BGN Biglycan
81	17.57575721	IPI00419585,9	PPIA Peptidyl-prolyl cis-trans isomerase A
81	13.00448477	IPI00887678,1	LOC654188 similar to peptidylprolyl isomerase A-like
82	5.454545468	IPI00002230,4	AADACL1 arylacetamide deacetylase-like 1
83	6.785980612	IPI00007334,1	ACIN1 Isoform 1 of Apoptotic chromatin condensation inducer in the nucleus
83	6.346483529	IPI00297991,2	ACIN1 Isoform 3 of Apoptotic chromatin condensation inducer in the nucleus
83	6.026058644	IPI00215975,1	ACIN1 Isoform 2 of Apoptotic chromatin condensation inducer in the nucleus
84	9.010989219	IPI00027107,5	TUFM Tu translation elongation factor, mitochondrial precursor
85	2.539145201	IPI00742682,2	TPR Nucleoprotein TPR
86	23.88059646	IPI00789389,1	SFRS10 8 kDa protein
86	19.79166716	IPI00872079,2	SFRS10 cDNA, FLJ96718, highly similar to Homo sapiens splicing factor, arginine/serine-rich 10 (transformer 2 homolog, Drosophila) (SFRS10), mRNA
86	19.79166716	IPI00301503,8	SFRS10 Isoform 1 of Splicing factor, arginine/serine-rich 10
86	19.72318292	IPI00555647,3	SFRS10 Splicing factor, arginine/serine-rich 10 (Transformer 2 homolog, Drosophila) variant
86	17.46031791	IPI00472633,5	SFRS10 cDNA FLJ40872 fis, clone TUTER2000283, highly similar to Homo sapiens transformer-2-beta (SFRS10) gene

87	7.766990364	IPI00453473,6	HIST1H4H;HIST2H4A;HIST4H4;HIST1H4F;HIST1H4D;HIST1H4K;HIST1H4C;HIST1H4J;HIST1H4A;HIST1H4I;HIST1H4B;HIST2H4B;HIST1H4E;HIST1H4L Histone H4
88	8.108107746	IPI00789511,2	SCPEP1 33 kDa protein
88	8.108107746	IPI00477895,3	SCPEP1 Isoform 2 of Retinoid-inducible serine carboxypeptidase
88	5.309734493	IPI00012426,1	SCPEP1 Isoform 1 of Retinoid-inducible serine carboxypeptidase
89	3.283815458	IPI00876952,1	- Putative uncharacterized protein ENSP00000370334
90	7.333333045	IPI00853047,1	NAMPT Putative uncharacterized protein NAMPT
90	7.236842066	IPI00853507,1	NAMPT Putative uncharacterized protein NAMPT
90	6.321839243	IPI00853098,1	NAMPT 21 kDa protein
90	5.295315757	IPI00018873,1	NAMPT Isoform 1 of Nicotinamide phosphoribosyltransferase
90	4.891304299	IPI00337370,3	NAMPT Isoform 2 of Nicotinamide phosphoribosyltransferase
91	3.479853645	IPI00441550,4	GLB1 Isoform 2 of Beta-galactosidase
91	2.93663051	IPI00797646,4	GLB1 galactosidase, beta 1 isoform b
91	2.806499228	IPI00441344,2	GLB1 Isoform 1 of Beta-galactosidase
92	6.870228797	IPI00003362,2	HSPA5 HSPA5 protein
93	10.27190313	IPI00299150,4	CTSS Cathepsin S
93	9.252668917	IPI00910216,1	- cDNA FLJ50259, highly similar to Cathepsin S
94	11.79940999	IPI00789310,1	TKT 37 kDa protein
94	11.17318422	IPI00793119,1	TKT cDNA FLJ32530 fis, clone SMINT2000185, highly similar to TRANSKETOLASE
94	9.629629552	IPI00792641,1	TKT transketolase isoform 2
94	8.240887523	IPI00643920,3	TKT cDNA FLJ54957, highly similar to Transketolase
95	4.918032885	IPI00917453,1	- cDNA FLJ40980 fis, clone UTERU2014464, highly similar to ACID CERAMIDASE
95	3.856041282	IPI00059685,6	ASAH1 N-acylsphingosine amidohydrolase 1 isoform c
95	3.797468171	IPI00013698,1	ASAH1 Acid ceramidase
95	3.649634868	IPI00418446,4	ASAH1 N-acylsphingosine amidohydrolase 1 isoform b
96	10.65573767	IPI00759832,1	YWHAB Isoform Short of 14-3-3 protein beta/alpha
96	10.56910604	IPI00216318,5	YWHAB Isoform Long of 14-3-3 protein beta/alpha
97	12.97071129	IPI00472610,2	IGHM IGHM protein
97	11.48936152	IPI00784810,1	IGHV4-31 IGHV4-31 protein
97	10.38135588	IPI00876888,1	- cDNA FLJ78387
97	10.31578928	IPI00807531,2	IGHG1 IGHG1 protein
97	7.796610147	IPI00892671,1	IGHG1 32 kDa protein
97	7.203389704	IPI00645363,2	IGHG1 Putative uncharacterized protein DKFZp686P15220

97	6.609195471	IPI00816681,1	IGHM Hepatitis B virus receptor binding protein (Fragment)
97	6.437768042	IPI00816314,1	IGHM Putative uncharacterized protein DKFZp686I15196
97	6.396588683	IPI00829944,1	IGHG1 IGHG1 protein
97	6.25	IPI00423463,1	IGHG1 Putative uncharacterized protein DKFZp686O01196
97	6.224066392	IPI00384938,1	IGHG1 Putative uncharacterized protein DKFZp686N02209
97	5.514705926	IPI00448925,3	IGHG1 IGHG1 protein
97	4.904051125	IPI00448938,1	IGHG1 IGHG1 protein
97	4.862579331	IPI00784822,1	IGHV4-31 IGHV4-31 protein
97	4.842105135	IPI00784842,1	IGHV4-31 Putative uncharacterized protein DKFZp686G11190
97	4.842105135	IPI00784817,1	IGHV4-31 Anti-RhD monoclonal T125 gamma1 heavy chain
97	4.842105135	IPI00423464,1	IGHG1 Putative uncharacterized protein DKFZp686K03196
97	4.831932858	IPI00785084,1	IGHV4-31 Immunoglobulin heavy variable 4-31
97	4.791666567	IPI00761159,1	IGHM IGHM protein
97	4.781704769	IPI00423466,1	IGHG1 Putative uncharacterized protein DKFZp686H20196
97	4.712812975	IPI00382606,1	F7 Factor VII active site mutant immunoconjugate
98	8.455882221	IPI00383227,1	- Calnexin (Fragment)
98	8.333333582	IPI00477719,5	CANX 32 kDa protein
98	3.668261692	IPI00020984,2	CANX cDNA FLJ55574, highly similar to Calnexin
99	7.602339238	IPI00220573,4	MRCL3 Myosin regulatory light chain MRLC3
99	7.558139414	IPI00719669,4	MRLC2 Myosin regulatory light chain
99	7.558139414	IPI00033494,3	MRLC2 Myosin regulatory light chain MRLC2
99	7.344632596	IPI00604523,1	MRCL3 Myosin regulatory light chain MRCL3 variant
100	13.54581714	IPI00413700,1	USF1 upstream stimulatory factor 1 isoform 2
100	12.05673739	IPI00514348,1	USF1 Upstream transcription factor 1
100	10.96774191	IPI00026559,1	USF1 Upstream stimulatory factor 1
101	6.666667014	IPI00005161,3	ARPC2 Actin-related protein 2/3 complex subunit 2
102	7.303370535	IPI00171626,3	LPCAT1 1-acylglycerophosphocholine O-acyltransferase 1
103	6.071428582	IPI00337754,3	PML Isoform PML-3B of Probable transcription factor PML
103	5.564648286	IPI00303999,1	PML Isoform PML-2 of Probable transcription factor PML
103	5.371247977	IPI00220453,1	PML Isoform PML-X of Probable transcription factor PML
103	5.304212123	IPI00744329,1	PML PML protein
103	4.126213491	IPI00304000,1	PML Isoform PML-3 of Probable transcription factor PML
103	4.101327062	IPI00332110,2	PML promyelocytic leukemia protein isoform 9

103	4.018912464	IPI00395707,2	PML promyelocytic leukemia protein isoform 7
103	3.981264681	IPI00382504,1	PML Tripartite motif protein TRIM19 delta
103	3.908045962	IPI00395893,5	PML promyelocytic leukemia protein isoform 8
103	3.854875267	IPI00022348,2	PML Isoform PML-1 of Probable transcription factor PML
103	2.905982919	IPI00181058,2	PML promyelocytic leukemia protein isoform 10
103	2.176696621	IPI00291097,8	PML promyelocytic leukemia protein isoform 11
103	2.132998779	IPI00876842,1	- PML-RAR protein
104	6.529209763	IPI00647597,2	DDX3Y cDNA FLJ59914, highly similar to ATP-dependent RNA helicase DDX3Y
104	5.303030461	IPI00293616,3	DDX3Y;LOC100130220 ATP-dependent RNA helicase DDX3Y
104	5.287009105	IPI00215637,5	DDX3X ATP-dependent RNA helicase DDX3X
104	4.135338217	IPI00910433,1	- cDNA FLJ60675, highly similar to ATP-dependent RNA helicase DDX3X
105	15.08620679	IPI00514106,3	LEMD2 Putative uncharacterized protein LEMD2
105	13.9165014	IPI00168336,1	LEMD2 LEM domain-containing protein 2
106	16.66666716	IPI00027463,1	S100A6 Protein S100-A6
107	32.32682049	IPI00789324,3	JUP cDNA FLJ60424, highly similar to Junction plakoglobin
107	30.23255765	IPI00789318,1	JUP 10 kDa protein
107	17.02127606	IPI00790671,1	JUP 26 kDa protein
107	14.44043368	IPI00788705,1	JUP 30 kDa protein
107	13.51351291	IPI00791432,1	JUP 32 kDa protein
107	5.369127542	IPI00554711,3	JUP Junction plakoglobin
108	14.38202262	IPI00007752,1	TUBB2C Tubulin beta-2C chain
108	12.43523285	IPI00908605,1	- cDNA FLJ59940, highly similar to Tubulin beta-2C chain
108	8.564231545	IPI00911016,1	- cDNA FLJ11352 fis, clone HEMBA1000020, highly similar to Tubulin beta-2C chain
109	5.211558193	IPI00024870,1	MYH11 smooth muscle myosin heavy chain 11 isoform SM2A
109	5.192802101	IPI00744256,1	MYH11 smooth muscle myosin heavy chain 11 isoform SM2B
109	5.121703818	IPI00020501,1	MYH11 Myosin-11
109	5.103587732	IPI00873792,1	MYH11 228 kDa protein
109	5.103587732	IPI00743857,1	MYH11 smooth muscle myosin heavy chain 11 isoform SM1B
109	4.977821559	IPI00873982,2	MYH11 Myosin heavy chain 11 smooth muscle isoform
110	14.03118074	IPI00026230,1	HNRNPH2 Heterogeneous nuclear ribonucleoprotein H2
110	11.63366362	IPI00909162,1	- cDNA FLJ57964, highly similar to Heterogeneous nuclear ribonucleoprotein H'
111	16.96428508	IPI00854592,2	TPM3 Tropomyosin 3
111	16.37931019	IPI00642042,3	TPM3 Putative uncharacterized protein DKFZp686J1372

111	15.38461596	IPI00477649,1	TPM3 tropomyosin 3 isoform 5
111	15.38461596	IPI00218320,4	TPM3 Isoform 3 of Tropomyosin alpha-3 chain
111	15.32258093	IPI00479185,1	TPM3 tropomyosin 3 isoform 4
111	15.32258093	IPI00382894,2	TPM3 Tropomyosin 3
111	15.32258093	IPI00218319,3	TPM3 Isoform 2 of Tropomyosin alpha-3 chain
111	9.122806787	IPI00183968,4	TPM3 tropomyosin 3 isoform 1
111	6.584361941	IPI00645055,3	TPM3 cDNA FLJ35371 fis, clone SKMUS2001740, highly similar to TROPOMYOSIN ALPHA CHAIN, SKELETAL MUSCLE
111	6.32911399	IPI00647245,1	TPM3 Tropomyosin 3
111	6.32911399	IPI00643370,1	TPM3 Tropomyosin 3
111	4.926108196	IPI00909961,1	- cDNA FLJ50720, highly similar to Homo sapiens tropomyosin 3 (TPM3), transcript variant 2, mRNA
112	13.08016926	IPI00797452,2	KRT4 cDNA FLJ55805, highly similar to Keratin, type II cytoskeletal 4
112	10.43771058	IPI00290078,5	KRT4 keratin 4
113	2.993051894	IPI00400922,5	PDCD11 Protein RRP5 homolog
114	5.66666685	IPI00871319,1	CYB5R3 34 kDa protein
114	5.647840351	IPI00328415,11	CYB5R3 Isoform 1 of NADH-cytochrome b5 reductase 3
114	4.316546768	IPI00446235,2	CYB5R3 Isoform 2 of NADH-cytochrome b5 reductase 3
115	10.6122449	IPI00021263,3	YWHAZ 14-3-3 protein zeta/delta
115	7.142857462	IPI00789337,2	YWHAZ cDNA, FLJ79516, highly similar to 14-3-3 protein zeta/delta
116	3.682170436	IPI00329791,9	DDX46 cDNA FLJ78679, highly similar to Homo sapiens DEAD (Asp-Glu-Ala-Asp) box polypeptide 46 (DDX46), mRNA
117	5.874263123	IPI00010951,2	EPPK1 epiplakin 1
118	6.995885074	IPI00903320,1	- cDNA FLJ36571 fis, clone TRACH2012242, highly similar to Myoferlin
118	2.872709185	IPI00216268,1	MYOF Isoform 2 of Myoferlin
118	2.83203125	IPI00645867,2	MYOF Isoform 6 of Myoferlin
118	2.827888913	IPI00216269,2	MYOF Isoform 3 of Myoferlin
118	2.814167924	IPI00021048,1	MYOF Isoform 1 of Myoferlin
119	6.041666493	IPI00552416,4	FLNA Filamin A, alpha
119	2.807775326	IPI00909642,1	- Filamin A
119	2.49328725	IPI00644576,1	FLNA Filamin A, alpha
119	2.463054098	IPI00302592,2	FLNA Isoform 2 of Filamin-A
119	2.455610037	IPI00333541,6	FLNA Isoform 1 of Filamin-A
120	5.978260934	IPI00019912,3	HSD17B4 Peroxisomal multifunctional enzyme type 2
120	5.781865865	IPI00909582,1	- cDNA FLJ55431, highly similar to Peroxisomal multifunctional enzyme type 2

120	5.077262595	IPI00847995,1	HSD17B4 Similar to Peroxisomal multifunctional protein 2
121	15.99999964	IPI00645957,1	PYHIN1 17 kDa protein
121	14.96063024	IPI00642094,1	IFI16 14 kDa protein
121	10.16949117	IPI00893471,1	PYHIN1 Isoform 6 of Pyrin and HIN domain-containing protein 1
121	9.795918316	IPI00103253,1	PYHIN1 Isoform 5 of Pyrin and HIN domain-containing protein 1
121	6.763284653	IPI00384836,2	IFI16 Isoform 4 of Gamma-interferon-inducible protein Ifi-16
121	5.309734493	IPI00397544,1	PYHIN1 Isoform 4 of Pyrin and HIN domain-containing protein 1
121	5.206073821	IPI00412281,1	PYHIN1 Isoform 3 of Pyrin and HIN domain-containing protein 1
121	4.968944192	IPI00397542,1	PYHIN1 Isoform 2 of Pyrin and HIN domain-containing protein 1
121	4.878048599	IPI00398847,1	PYHIN1 Isoform 1 of Pyrin and HIN domain-containing protein 1
121	4.160475358	IPI00217475,2	IFI16 Isoform 3 of Gamma-interferon-inducible protein Ifi-16
121	3.840877861	IPI00217474,5	IFI16 Isoform 2 of Gamma-interferon-inducible protein Ifi-16
121	3.81991826	IPI00909417,1	- cDNA FLJ61295, highly similar to Gamma-interferon-inducible protein Ifi-16
121	3.81991826	IPI00643081,3	IFI16 Gamma-interferon-inducible protein Ifi-16
121	3.566879034	IPI00003443,3	IFI16 Isoform 1 of Gamma-interferon-inducible protein Ifi-16
122	6.904762238	IPI00879638,2	DDX17 cDNA FLJ58652, highly similar to Probable ATP-dependent RNA helicase DDX17
122	6.72153607	IPI00023785,6	DDX17 DEAD box polypeptide 17 isoform 1
122	5.521472543	IPI00651653,1	DDX17 Isoform 3 of Probable ATP-dependent RNA helicase DDX17
122	4.44785282	IPI00889541,1	DDX17 DEAD (Asp-Glu-Ala-Asp) box polypeptide 17
122	4.44785282	IPI00651677,1	DDX17 Isoform 2 of Probable ATP-dependent RNA helicase DDX17
123	5.622490123	IPI00456887,2	HNRNPUL2 Heterogeneous nuclear ribonucleoprotein U-like protein 2
124	8.108107746	IPI00414963,2	PABPN1 Isoform 2 of Polyadenylate-binding protein 2
124	7.843137532	IPI00005792,2	PABPN1 Isoform 1 of Polyadenylate-binding protein 2
125	4.039497301	IPI00384542,3	NID1 Isoform 2 of Nidogen-1
125	3.608660772	IPI00026944,2	NID1 Isoform 1 of Nidogen-1
126	3.506907448	IPI00305068,5	PRPF6 Pre-mRNA-processing factor 6
127	8.431372792	IPI00296053,3	FH Isoform Mitochondrial of Fumarate hydratase, mitochondrial
127	7.066380978	IPI00759715,1	FH Isoform Cytoplasmic of Fumarate hydratase, mitochondrial
128	7.52864182	IPI00219077,4	LTA4H Isoform 1 of Leukotriene A-4 hydrolase
128	7.086614519	IPI00793812,2	LTA4H cDNA FLJ51009, highly similar to Leukotriene A-4 hydrolase
128	6.703910977	IPI00514090,2	LTA4H Isoform 2 of Leukotriene A-4 hydrolase
128	4.436860234	IPI00794758,1	LTA4H 32 kDa protein
129	7.623318583	IPI00013174,2	RBM14;RBM4 Isoform 1 of RNA-binding protein 14

130	13.69294673	IPI00917430,1	SF3B1 Protein
130	2.530674823	IPI00026089,3	SF3B1 Splicing factor 3B subunit 1
131	13.60000074	IPI00909939,1	- cDNA FLJ52195, highly similar to LIM and SH3 domain protein 1
131	12.65060306	IPI00883946,1	LASP1 19 kDa protein
131	12.27272749	IPI00909481,1	- cDNA FLJ58065, highly similar to LIM and SH3 domain protein 1
131	10.24390236	IPI00909678,1	- cDNA FLJ51834, highly similar to LIM and SH3 domain protein 1
131	8.045977354	IPI00000861,1	LASP1 Isoform 1 of LIM and SH3 domain protein 1
131	6.501547992	IPI00386803,4	LASP1 Isoform 2 of LIM and SH3 domain protein 1
132	2.876869962	IPI00646947,2	ATP2A1 cDNA FLJ46599 fis, clone THYMU3047115, highly similar to Sarcoplasmic/endoplasmic reticulum calcium
132	2.515090629	IPI00396118,3	ATP2A1 Isoform SERCA1A of Sarcoplasmic/endoplasmic reticulum calcium ATPase 1
132	2.497502416	IPI00024804,3	ATP2A1 Isoform SERCA1B of Sarcoplasmic/endoplasmic reticulum calcium ATPase 1
132	2.220166475	IPI00871296,1	ATP2A3 118 kDa protein
132	1.302605215	IPI00478023,1	ATP2A3 Isoform SERCA3D of Sarcoplasmic/endoplasmic reticulum calcium ATPase 3
132	1.301301271	IPI00748794,1	ATP2A3 Isoform SERCA3A of Sarcoplasmic/endoplasmic reticulum calcium ATPase 3
132	1.263362449	IPI00218440,1	ATP2A3 Isoform SERCA3C of Sarcoplasmic/endoplasmic reticulum calcium ATPase 3
132	1.259689964	IPI00871568,1	ATP2A3 Sarco/endoplasmic reticulum Ca2+ ATPase isoform 3f
132	1.246404648	IPI00004092,2	ATP2A3 Isoform SERCA3B of Sarcoplasmic/endoplasmic reticulum calcium ATPase 3
132	1.23574147	IPI00303760,2	ATP2A3 ATPase, Ca++ transporting, ubiquitous isoform e
132	1.23574147	IPI00218442,4	ATP2A3 Putative uncharacterized protein ATP2A3
132	0.947867334	IPI00872163,1	ATP2A1 Similar to ATPase, Ca++ transporting, cardiac muscle, fast twitch 1 (Fragment)
132	0.802407227	IPI00747443,2	ATP2A2 Putative uncharacterized protein ATP2A2
132	0.802407227	IPI00177817,4	ATP2A2 Isoform SERCA2A of Sarcoplasmic/endoplasmic reticulum calcium ATPase 2
132	0.788177364	IPI00914019,1	ATP2A2 ATPase, Ca++ transporting, slow twitch 2 isoform 3
132	0.767754298	IPI00219078,5	ATP2A2 Isoform SERCA2B of Sarcoplasmic/endoplasmic reticulum calcium ATPase 2
133	5.795314535	IPI00852806,1	EXOC6B SEC15-like 2
133	4.552129284	IPI00917263,1	EXOC6B Exocyst complex component 6B
134	16.02209955	IPI00798089,1	RAB2A 21 kDa protein
134	7.428571582	IPI00794027,1	RAB2A Protein
134	6.132075563	IPI00031169,1	RAB2A Ras-related protein Rab-2A
134	6.10328652	IPI00873632,1	RAB2A 24 kDa protein
134	6.018518656	IPI00102896,1	RAB2B Ras-related protein Rab-2B
135	10.71428582	IPI00465439,5	ALDOA Fructose-bisphosphate aldolase A
135	9.33014378	IPI00796333,1	ALDOA 45 kDa protein

136	10.25641039	IPI00607772,1	ARPC4 actin related protein 2/3 complex subunit 4 isoform b
136	4.761904851	IPI00554811,2	TTLL3;ARPC4 Actin-related protein 2/3 complex subunit 4
136	2.879999951	IPI00790262,1	TTLL3 Similar to Actin-related protein 2/3 complex subunit 4
137	8.902077377	IPI00744692,1	TALDO1 Transaldolase
138	6.535948068	IPI00644329,1	SMARCA2 SWI/SNF related, matrix associated, actin dependent regulator of chromatin, subfamily a, member 2
138	5.84795326	IPI00643691,1	SMARCA2 SWI/SNF related, matrix associated, actin dependent regulator of chromatin, subfamily a, member 2
138	5.649717525	IPI00645885,1	SMARCA2 SWI/SNF related, matrix associated, actin dependent regulator of chromatin, subfamily a, member 2
138	4.237288237	IPI00642503,1	SMARCA2 SWI/SNF related, matrix associated, actin dependent regulator of chromatin, subfamily a, member 2
138	3.937007859	IPI00103451,3	SMARCA2 SWI/SNF related, matrix associated, actin dependent regulator of chromatin, subfamily a, member 2
138	3.690036759	IPI00640994,1	SMARCA2 SWI/SNF related, matrix associated, actin dependent regulator of chromatin, subfamily a, member 2
138	3.597122431	IPI00647560,1	SMARCA2 cDNA FLJ36757 fis, clone UTERU2018522, highly similar to Human transcriptional activator hSNF2a
138	2.417302877	IPI00386718,4	SMARCA2 Isoform Short of Probable global transcription activator SNF2L2
138	2.38993708	IPI00514648,1	SMARCA2 Isoform Long of Probable global transcription activator SNF2L2
138	1.487910748	IPI00900328,1	SMARCA4 SWI/SNF-related matrix-associated actin-dependent regulator of chromatin a4 isoform F
138	1.486988831	IPI00900338,1	SMARCA4 SWI/SNF-related matrix-associated actin-dependent regulator of chromatin a4 isoform E
138	1.484230068	IPI00900285,1	SMARCA4 SWI/SNF-related matrix-associated actin-dependent regulator of chromatin a4 isoform C
138	1.457194891	IPI00293426,2	SMARCA4 Probable global transcription activator SNF2L4
138	1.430274174	IPI00029822,4	SMARCA4 SWI/SNF-related matrix-associated actin-dependent regulator of chromatin a4 isoform D
138	1.429422293	IPI00900269,1	SMARCA4 SWI/SNF-related matrix-associated actin-dependent regulator of chromatin a4 isoform A
139	3.200883046	IPI00215948,4	CTNNA1 Isoform 1 of Catenin alpha-1
139	3.118279576	IPI00473136,3	CTNNA1 Isoform 2 of Catenin alpha-1
140	4.780876637	IPI00438230,3	TRIM28 Isoform 2 of Transcription intermediary factor 1-beta
140	4.311377183	IPI00438229,2	TRIM28 Isoform 1 of Transcription intermediary factor 1-beta
141	3.386960179	IPI00025058,2	ADAR Isoform 3 of Double-stranded RNA-specific adenosine deaminase
141	3.333333507	IPI00025057,2	ADAR Isoform 2 of Double-stranded RNA-specific adenosine deaminase
141	3.262642771	IPI00394665,4	ADAR Isoform 1 of Double-stranded RNA-specific adenosine deaminase
141	3.152088076	IPI00394668,2	ADAR Isoform 4 of Double-stranded RNA-specific adenosine deaminase
141	3.007518873	IPI00760588,2	ADAR Isoform 5 of Double-stranded RNA-specific adenosine deaminase
142	14.81481493	IPI00791498,1	ATP5B 17 kDa protein
142	8.128544688	IPI00303476,1	ATP5B ATP synthase subunit beta, mitochondrial
143	2.297998592	IPI00291802,3	LMO7 Isoform 3 of LIM domain only protein 7
144	4.088397697	IPI00220667,3	HK1 Isoform 4 of Hexokinase-1
144	3.275109082	IPI00220663,3	HK1 Isoform 2 of Hexokinase-1

144	3.271537647	IPI00018246,5	HK1 Isoform 1 of Hexokinase-1
144	3.257329017	IPI00220665,6	HK1 Isoform 3 of Hexokinase-1
144	3.151260689	IPI00903226,1	- cDNA FLJ46359 fis, clone TESTI4049786, highly similar to Hexokinase-1
145	4.29338105	IPI00219825,2	PSAP Prosaposin
145	3.750000149	IPI00556640,1	PSAP PSAP protein
145	3.625954315	IPI00012503,1	PSAP Isoform Sap-mu-0 of Proactivator polypeptide
145	3.612167388	IPI00873201,1	PSAP Isoform Sap-mu-6 of Proactivator polypeptide
145	3.605313227	IPI00873020,1	PSAP Prosaposin variant
145	3.605313227	IPI00744835,1	PSAP Isoform Sap-mu-9 of Proactivator polypeptide
146	4.418262094	IPI00178744,4	ACADVL Isoform 2 of Very long-chain specific acyl-CoA dehydrogenase, mitochondrial
146	4.279600456	IPI00028031,2	ACADVL cDNA FLJ56425, highly similar to Very-long-chain specific acyl-CoAdehydrogenase, mitochondrial
147	8.799999952	IPI00902755,1	FGA FGA protein (Fragment)
147	3.669724613	IPI00877029,1	FGA FGA protein
147	3.233256191	IPI00021885,1	FGA Isoform 1 of Fibrinogen alpha chain
147	2.768166177	IPI00871469,1	FGA FGA protein
147	2.639751509	IPI00029717,1	FGA Isoform 2 of Fibrinogen alpha chain
148	4.914933816	IPI00006379,1	NOP5/NOP58 Nucleolar protein 5
149	6.42201826	IPI00916818,1	- cDNA FLJ53125, highly similar to Phosphoglycerate kinase 1
149	5.035971105	IPI00219568,4	PGK2 Phosphoglycerate kinase 2
149	5.035971105	IPI00169383,3	PGK1 Phosphoglycerate kinase 1
150	5.000000075	IPI00642732,1	PDHA1 Pyruvate dehydrogenase (Lipoamide) alpha 1
150	4.615384713	IPI00916629,1	PDHA1 Pyruvate dehydrogenase E1 component subunit alpha, somatic form, mitochondrial
150	4.411764815	IPI00643575,1	PDHA1 Pyruvate dehydrogenase (Lipoamide) alpha 1
150	4.205607623	IPI00306301,2	PDHA1 Mitochondrial PDHA1
151	3.358209133	IPI00013830,1	SNW1 SNW domain-containing protein 1
151	3.15236412	IPI00910816,1	- cDNA FLJ59238, highly similar to SNW domain-containing protein 1
152	17.46031791	IPI00893767,1	C22orf28 7 kDa protein
152	10.89743599	IPI00910259,1	- cDNA FLJ57223
152	9.714286029	IPI00909218,1	- cDNA FLJ57752
152	4.883721098	IPI00910144,1	- cDNA FLJ58027
152	3.366336599	IPI00550689,3	C22orf28 UPF0027 protein C22orf28
153	5.960264802	IPI00854677,1	FUS Putative uncharacterized protein FUS
153	5.732484162	IPI00428056,1	- BBF2H7/FUS protein (Fragment)

153	4.89510484	IPI00909890,1	- cDNA FLJ58049, highly similar to RNA-binding protein FUS
153	4.022988677	IPI00645208,3	FUS 53 kDa protein
153	3.999999911	IPI00221354,1	FUS Isoform Short of RNA-binding protein FUS
153	3.97727266	IPI00260715,5	FUS Fus-like protein (Fragment)
153	1.528013591	IPI00020194,1	TAF15 Isoform Short of TATA-binding protein-associated factor 2N
153	1.520270295	IPI00294426,3	TAF15 Isoform Long of TATA-binding protein-associated factor 2N
153	1.446945313	IPI00873762,1	TAF15 65 kDa protein
154	6.593406945	IPI00909303,1	- cDNA FLJ58073, moderately similar to Cathepsin B
154	6.521739066	IPI00903045,1	- cDNA FLJ40065 fis, clone TESOP2000400, highly similar to CATHEPSIN B
154	5.309734493	IPI00295741,4	CTSB Cathepsin B
155	3.225806355	IPI00909237,1	- cDNA FLJ55703, highly similar to Solute carrier family 2, facilitated glucose transporter member 1
155	2.845528536	IPI00872375,2	SLC2A1 Putative uncharacterized protein SLC2A1 (Fragment)
155	2.845528536	IPI00220194,6	SLC2A1 Solute carrier family 2, facilitated glucose transporter member 1
156	9.417040646	IPI00908538,1	- cDNA FLJ54293
156	7.692307979	IPI00844406,1	ARGLU1 Isoform 2 of Arginine and glutamate-rich protein 1
156	7.692307979	IPI00478834,3	ARGLU1 Isoform 1 of Arginine and glutamate-rich protein 1
157	3.448275849	IPI00908488,1	- cDNA FLJ60932, highly similar to T-complex protein 1 subunit zeta-2
157	3.207547218	IPI00220656,4	CCT6B T-complex protein 1 subunit zeta-2
157	3.201506659	IPI00027626,3	CCT6A T-complex protein 1 subunit zeta
158	1.417769399	IPI00902463,1	- cDNA FLJ46898 fis, clone UTERU3022168, highly similar to Protein FAM62A
158	1.358695608	IPI00022143,3	FAM62A Isoform 1 of Extended synaptotagmin-1
158	1.3464991	IPI00746655,1	FAM62A Isoform 2 of Extended synaptotagmin-1
159	12.22222224	IPI00893518,1	- Flotillin 1
159	5.913978443	IPI00894029,1	FLOT1 Flotillin 1
159	4.918032885	IPI00607626,1	FLOT1 Flotillin 1
159	3.957783803	IPI00892872,2	- cDNA FLJ54943, highly similar to Flotillin-1
159	3.512880579	IPI00027438,2	FLOT1 Flotillin-1
160	18.36734712	IPI00790580,1	RPSA 16 kDa protein
160	9.246575087	IPI00399077,4	LOC388122 hypothetical protein
160	9.152542055	IPI00887924,1	LOC387867 similar to Ribosomal protein SA pseudogene isoform 1
160	9.152542055	IPI00847766,1	LOC388524 Similar to Ribosomal protein 40
160	9.152542055	IPI00793137,2	- Putative uncharacterized protein ENSP00000346598
160	9.152542055	IPI00553164,4	RPSA 40S ribosomal protein SA

160	9.152542055	IPI00411639,1	RPSAP15 Laminin receptor-like protein LAMRL5
160	9.152542055	IPI00399036,1	hCG_1984468 hypothetical protein LOC389672
160	9.152542055	IPI00398958,3	LOC387867 similar to 40S ribosomal protein SA (p40) (34/67 kDa laminin receptor) (Colon carcinoma laminin-binding protein) (NEM/1CHD4) (Multidrug resistance-associated protein MGr1-Ag) isoform 1
160	9.000000358	IPI00413108,4	RPSA 33 kDa protein
160	7.981220633	IPI00793905,1	RPSA 24 kDa protein
160	6.538461894	IPI00889080,1	RP11-556K13,1 similar to hCG1997137 isoform 1
160	5.84192425	IPI00873552,1	- Putative uncharacterized protein ENSP00000381679 (Fragment)
160	5.7823129	IPI00745789,2	RP11-556K13,1 similar to 40S ribosomal protein SA (p40) (34/67 kDa laminin receptor) (Colon carcinoma laminin-binding protein) (NEM/1CHD4) (Multidrug resistance-associated protein MGr1-Ag) isoform 1
161	2.800000086	IPI00640106,2	HNRNPU cDNA FLJ44920 fis, clone BRAMY3011501, highly similar to Heterogeneous nuclear ribonucleoprotein U
161	2.678571455	IPI00644224,2	- cDNA FLJ54020, highly similar to Heterogeneous nuclear ribonucleoprotein U
161	2.605459094	IPI00479217,1	HNRNPU Isoform Short of Heterogeneous nuclear ribonucleoprotein U
161	2.548543736	IPI00883857,1	HNRNPU Isoform Long of Heterogeneous nuclear ribonucleoprotein U
161	2.545454539	IPI00644079,2	HNRNPU heterogeneous nuclear ribonucleoprotein U isoform a
162	9.595959634	IPI00877626,1	UNC84B Unc-84 homolog B
162	3.387533873	IPI00878697,1	UNC84B UNC84B protein
162	3.324468061	IPI00295940,4	UNC84B cDNA FLJ55508, highly similar to Sad1/unc-84-like protein 2
163	15.96638709	IPI00877625,1	FGG 14 kDa protein
163	15.44715464	IPI00411626,4	FGG Putative uncharacterized protein DKFZp779N0926
163	4.347826168	IPI00219713,1	FGG Isoform Gamma-A of Fibrinogen gamma chain
163	4.269662872	IPI00877792,1	FGG 50 kDa protein
163	4.194260389	IPI00021891,5	FGG Isoform Gamma-B of Fibrinogen gamma chain
163	4.121474922	IPI00877703,1	FGG 52 kDa protein
164	13.06532621	IPI00607820,1	GAR1 Isoform 2 of H/ACA ribonucleoprotein complex subunit 1
164	11.981567	IPI00302176,5	GAR1 Isoform 1 of H/ACA ribonucleoprotein complex subunit 1
164	10.48387066	IPI00872985,1	GAR1 26 kDa protein
165	7.999999821	IPI00894333,1	PPP1CB 14 kDa protein
165	7.246376574	IPI00892668,1	PPP1CB 16 kDa protein
165	5.95238097	IPI00788843,1	PPP3CC 19 kDa protein
165	5.91715984	IPI00892511,1	PPP1CB 20 kDa protein
165	5.586592108	IPI00894274,2	PPP1CB cDNA FLJ58972, highly similar to Serine/threonine-protein phosphatase PP1-beta catalytic subunit
165	4.233871028	IPI00748557,1	PPP3CB Serine/threonine protein phosphatase

165	4.085602984	IPI00218862,1	PPP3CB Isoform 2 of Serine/threonine-protein phosphatase 2B catalytic subunit beta isoform
165	4.077669978	IPI00181738,3	PPP3CB protein phosphatase 3 (formerly 2B), catalytic subunit, beta isoform isoform c
165	4.007633403	IPI00872930,1	PPP3CB Isoform 1 of Serine/threonine-protein phosphatase 2B catalytic subunit beta isoform
165	3.999999911	IPI00027809,2	PPP3CB protein phosphatase 3 (formerly 2B), catalytic subunit, beta isoform isoform a
165	3.585657477	IPI00305491,5	PPP3CC Serine/threonine-protein phosphatase 2B catalytic subunit gamma isoform
165	3.515625	IPI00413731,3	PPP3CC Serine/threonine protein phosphatase
165	3.496503457	IPI00410128,2	PPP1CA protein phosphatase 1, catalytic subunit, alpha isoform 2
165	3.454894572	IPI00872489,2	PPP3CC cDNA FLJ58322, highly similar to Serine/threonine-protein phosphatase 2B catalytic subunit gamma isoform
165	3.095975146	IPI00005705,1	PPP1CC Isoform Gamma-1 of Serine/threonine-protein phosphatase PP1-gamma catalytic subunit
165	3.058104031	IPI00218236,6	PPP1CB Serine/threonine-protein phosphatase PP1-beta catalytic subunit
165	3.030303121	IPI00550451,1	PPP1CA Serine/threonine-protein phosphatase PP1-alpha catalytic subunit
165	2.967359126	IPI00218187,1	PPP1CC Isoform Gamma-2 of Serine/threonine-protein phosphatase PP1-gamma catalytic subunit
165	2.932551317	IPI00027423,3	PPP1CA protein phosphatase 1, catalytic subunit, alpha isoform 3
165	2.808988839	IPI00871289,1	PPP1CB 40 kDa protein
165	2.724795602	IPI00872177,1	PPP1CB 41 kDa protein
165	2.421307564	IPI00902512,1	- cDNA FLJ45714 fis, clone FEKID2002637, highly similar to Serine/threonine-protein phosphatase PP1-alphacatalytic
165	2.132196166	IPI00910825,1	PPP3CA protein phosphatase 3, catalytic subunit, alpha isoform isoform 3
165	1.956947148	IPI00747748,1	PPP3CA Isoform 2 of Serine/threonine-protein phosphatase 2B catalytic subunit alpha isoform
165	1.919385791	IPI00179415,4	PPP3CA Isoform 1 of Serine/threonine-protein phosphatase 2B catalytic subunit alpha isoform
165	1.769911498	IPI00872209,1	PPP3CA 63 kDa protein
166	11.39705852	IPI00871908,2	- Putative uncharacterized protein ENSP00000260536 (Fragment)
166	4.807692394	IPI00794746,1	RPL7 Putative uncharacterized protein RPL7
166	4.524886981	IPI00872697,1	- Putative uncharacterized protein ENSP00000382847 (Fragment)
166	4.065040499	IPI00878826,1	- 29 kDa protein
166	4.048582911	IPI00796861,1	LOC100130892 WUGSC:H_RG054D04,1 protein
166	4.032257944	IPI00030179,3	RPL7 60S ribosomal protein L7
166	3.861003742	IPI00472171,3	RPL7 30 kDa protein
166	3.861003742	IPI00144171,2	hCG_2015956 hypothetical protein LOC648000
166	3.676470742	IPI00871827,1	RPL7 Putative uncharacterized protein RPL7 (Fragment)
166	3.663003817	IPI00872387,1	- Putative uncharacterized protein ENSP00000381447 (Fragment)
167	3.683241084	IPI00871856,2	STIP1 cDNA FLJ76863, highly similar to Homo sapiens stress-induced-phosphoprotein 1 (Hsp70/Hsp90-organizing
167	3.683241084	IPI00013894,1	STIP1 Stress-induced-phosphoprotein 1

168	11.47540957	IPI00607577,2	SOD2 manganese superoxide dismutase isoform B precursor
168	9.459459782	IPI00847322,1	SOD2 manganese superoxide dismutase isoform A precursor
168	3.97727266	IPI00896370,2	SOD2 cDNA FLJ40076 fis, clone TESTI2000874, highly similar to Superoxide dismutase
168	3.153153136	IPI00022314,1	SOD2 Superoxide dismutase [Mn], mitochondrial
169	6.944444776	IPI00643041,3	RAN GTP-binding nuclear protein Ran
169	6.437768042	IPI00795671,1	RAN RAN, member RAS oncogene family, isoform CRA_c
169	6.410256773	IPI00796462,1	RAN 27 kDa protein
169	6.355932355	IPI00792352,1	RAN 26 kDa protein
170	20.0000003	IPI00793089,1	MYL6B 13 kDa protein
170	15.86206853	IPI00796366,1	MYL6B 16 kDa protein
170	15.23178816	IPI00789605,1	MYL6;MYL6B Isoform Smooth muscle of Myosin light polypeptide 6
170	15.23178816	IPI00335168,9	MYL6B;MYL6 Isoform Non-muscle of Myosin light polypeptide 6
170	5.29801324	IPI00101961,2	- 17 kDa protein
171	23.00885022	IPI00790745,1	TFRC 13 kDa protein
171	3.421052545	IPI00022462,2	TFRC Transferrin receptor protein 1
172	7.62463361	IPI00759806,1	ENO1 Isoform MBP-1 of Alpha-enolase
172	5.990783498	IPI00465248,5	ENO1 Isoform alpha-enolase of Alpha-enolase
173	6.396588683	IPI00646512,1	RBBP7 Retinoblastoma binding protein 7
173	5.909090862	IPI00645757,1	RBBP4 Protein
173	3.333333507	IPI00877802,2	RBBP4 retinoblastoma binding protein 4 isoform c
173	3.170731664	IPI00645329,1	RBBP4 46 kDa protein
173	3.170731664	IPI00552530,2	RBBP7 Retinoblastoma binding protein 7
173	3.16301696	IPI00879702,1	RBBP7 Retinoblastoma binding protein 7
173	3.066037782	IPI00877934,1	RBBP4 retinoblastoma binding protein 4 isoform b
173	3.058823571	IPI00395865,4	RBBP7 Histone-binding protein RBBP7
173	3.058823571	IPI00328319,8	RBBP4 Histone-binding protein RBBP4
174	14.36464041	IPI00641351,1	RALY RNA binding protein, autoantigenic
174	12.3595506	IPI00640938,1	RALY RNA binding protein, autoantigenic
174	8.469055593	IPI00011268,2	RALY RNA binding protein, autoantigenic (HnRNP-associated with lethal yellow homolog (Mouse)), isoform CRA_a (Fragment)
174	4.641350359	IPI00642213,1	RALY RNA binding protein, autoantigenic
174	3.79310362	IPI00216044,1	RALY Isoform 1 of RNA-binding protein Raly
175	3.623188287	IPI00219330,2	ILF3 Isoform 5 of Interleukin enhancer-binding factor 3

175	3.561253473	IPI00298789,2	ILF3 Isoform 2 of Interleukin enhancer-binding factor 3
175	3.541076556	IPI00556173,1	ILF3 Isoform 6 of Interleukin enhancer-binding factor 3
175	3.272251412	IPI00414335,2	ILF3 Isoform 3 of Interleukin enhancer-binding factor 3
175	2.796420641	IPI00298788,4	ILF3 Isoform 1 of Interleukin enhancer-binding factor 3
175	2.783964388	IPI00418313,3	ILF3 interleukin enhancer binding factor 3 isoform d
176	0.795880146	IPI00420014,2	SNRNP200 Isoform 1 of U5 small nuclear ribonucleoprotein 200 kDa helicase
176	0.764705893	IPI00740142,2	LOC652147 hypothetical protein, partial
177	5.970149115	IPI00394820,3	OLFML1 Olfactomedin-like protein 1
178	13.51351291	IPI00386318,3	NECAP2 cDNA FLJ52763, highly similar to Adaptin ear-binding coat-associated protein 2
178	9.5057033	IPI00018188,3	NECAP2 Isoform 1 of Adaptin ear-binding coat-associated protein 2
178	9.157509357	IPI00073436,3	NECAP2 Isoform 2 of Adaptin ear-binding coat-associated protein 2
179	7.2580643	IPI00646520,1	NONO 15 kDa protein
179	4.883227125	IPI00304596,3	NONO Non-POU domain-containing octamer-binding protein
179	4.347826168	IPI00645966,1	NONO 24 kDa protein
179	3.62903215	IPI00645010,1	NONO 30 kDa protein
180	11.61971837	IPI00304435,3	NIPSNAP1 Protein NipSnap homolog 1
181	2.380952425	IPI00217563,4	ITGB1 Isoform Beta-1A of Integrin beta-1
182	9.268292785	IPI00025512,2	HSPB1 Heat shock protein beta-1
182	5.294117704	IPI00909453,1	- cDNA FLJ52243, highly similar to Heat-shock protein beta-1
183	3.862661123	IPI00025366,4	CS Citrate synthase, mitochondrial
183	3.296703473	IPI00796979,1	CS 30 kDa protein
184	1.851851866	IPI00019580,1	PLG Plasminogen
185	6.217616424	IPI00018236,2	GM2A Ganglioside GM2 activator
186	4.319371656	IPI00004573,2	PIGR Polymeric immunoglobulin receptor
187	2.283105068	IPI00001639,2	KPNB1 Importin subunit beta-1
188	3.806228191	IPI00910950,1	- cDNA FLJ51182, highly similar to 6-phosphogluconate dehydrogenase, decarboxylating
188	2.277432755	IPI00219525,10	PGD 6-phosphogluconate dehydrogenase, decarboxylating
188	2.140077762	IPI00747533,2	PGD 56 kDa protein
189	4.675324634	IPI00554617,2	TPP1 cDNA FLJ57277, highly similar to Tripeptidyl-peptidase 1
189	3.62903215	IPI00909516,1	- cDNA FLJ58558, highly similar to Tripeptidyl-peptidase 1
189	3.243243322	IPI00554538,5	TPP1 Putative uncharacterized protein TPP1
189	3.146853298	IPI00298237,8	TPP1 cDNA FLJ56402, highly similar to Tripeptidyl-peptidase 1
190	9.489051253	IPI00908950,1	- cDNA FLJ59178, moderately similar to 60S ribosomal protein L18

190	6.914893538	IPI00215719,6	RPL18 60S ribosomal protein L18
191	7.874015719	IPI00796965,1	NAP1L1 15 kDa protein
191	5.617977679	IPI00788840,1	NAP1L4 20 kDa protein
191	5.102040991	IPI00798071,1	NAP1L4 Putative uncharacterized protein NAP1L4
191	3.597122431	IPI00789437,1	NAP1L4 32 kDa protein
191	3.095975146	IPI00902909,1	- Nucleosome assembly protein 1-like 1, isoform CRA_c
191	2.717391215	IPI00789029,1	NAP1L1 cDNA FLJ30458 fis, clone BRACE2009421, highly similar to NUCLEOSOME ASSEMBLY PROTEIN 1-LIKE 1
191	2.590673603	IPI00017763,6	NAP1L4 cDNA FLJ59403, highly similar to Nucleosome assembly protein 1-like 4
191	2.557544783	IPI00023860,1	NAP1L1 Nucleosome assembly protein 1-like 1
192	4.982206225	IPI00902867,1	HPR cDNA FLJ31310 fis, clone LIVER1000165, highly similar to Haptoglobin
192	4.982206225	IPI00431645,1	HP HP protein
192	4.034582153	IPI00478493,3	HP;HPR haptoglobin isoform 2 preproprotein
192	3.448275849	IPI00902590,1	HP Haptoglobin
192	3.333333507	IPI00641737,1	HPR 47 kDa protein
193	4.347826168	IPI00878947,1	HP1BP3 Heterochromatin protein 1, binding protein 3
193	3.956834599	IPI00640417,1	HP1BP3 Heterochromatin protein 1, binding protein 3
193	3.197674453	IPI00645339,2	HP1BP3 Isoform 4 of Heterochromatin protein 1-binding protein 3
193	2.743142098	IPI00871239,1	HP1BP3 Isoform 3 of Heterochromatin protein 1-binding protein 3
193	2.135922387	IPI00646486,1	HP1BP3 Isoform 2 of Heterochromatin protein 1-binding protein 3
193	1.989150047	IPI00642238,4	HP1BP3 Isoform 1 of Heterochromatin protein 1-binding protein 3
194	4.918032885	IPI00878140,3	TST cDNA FLJ57870, highly similar to Thiosulfate sulfurtransferase
194	4.040404037	IPI00216293,6	TST Thiosulfate sulfurtransferase
195	4.095563293	IPI00877025,1	MPO Putative uncharacterized protein MPO (Fragment)
195	1.846153848	IPI00236554,1	MPO Isoform H14 of Myeloperoxidase
195	1.610738225	IPI00007244,1	MPO Isoform H17 of Myeloperoxidase
195	1.544401515	IPI00236556,1	MPO Isoform H7 of Myeloperoxidase
196	4.729729891	IPI00872758,1	TOR1AIP1 Torsin A interacting protein 1
196	3.341288865	IPI00644766,4	TOR1AIP1 Similar to Torsin-1A-interacting protein 1
196	2.397260256	IPI00792065,3	TOR1AIP1 Putative uncharacterized protein TOR1AIP1
197	8.196721226	IPI00376039,3	LOC388076 Putative uncharacterized protein ENSP00000385291
197	7.978723198	IPI00645201,1	RPS8 Ribosomal protein S8
197	7.211538404	IPI00216587,9	RPS8 40S ribosomal protein S8

197	6.912442297	IPI00872430,1	RPS8 25 kDa protein
198	2.325581387	IPI00871221,1	ATP1B1 Isoform 2 of Sodium/potassium-transporting ATPase subunit beta-1
198	2.310230955	IPI00747849,2	ATP1B1 Isoform 1 of Sodium/potassium-transporting ATPase subunit beta-1
199	4.048582911	IPI00004845,4	NIPSNAP3A Protein NipSnap homolog 3A
199	3.533568978	IPI00871176,1	NIPSNAP3A 32 kDa protein
200	6.060606241	IPI00847579,1	RPS12 ribosomal protein S12
200	6.060606241	IPI00737299,1	LOC391370 similar to hCG1818387
200	6.060606241	IPI00376429,3	LOC391370 similar to hCG1818387
200	6.060606241	IPI00013917,2	RPS12 40S ribosomal protein S12
200	6.015037745	IPI00157456,1	LOC727997 similar to 40S ribosomal protein S12
201	7.182320207	IPI00829824,1	- PRKCSH protein (Fragment)
201	2.476190403	IPI00792916,2	PRKCSH protein kinase C substrate 80K-H isoform 2
201	2.429906465	IPI00026154,3	PRKCSH cDNA FLJ59211, highly similar to Glucosidase 2 subunit beta
202	9.677419066	IPI00815843,1	- RPL14 protein (Fragment)
202	5.633802712	IPI00002821,4	RPL14 60S ribosomal protein L14
202	5.581395328	IPI00069693,4	- Putative uncharacterized protein RPL14L
202	5.454545468	IPI00555744,6	RPL14 Ribosomal protein L14 variant
203	7.079645991	IPI00029740,2	SNX3 Isoform 3 of Sorting nexin-3
203	5.714285746	IPI00552276,5	SNX3 Isoform 4 of Sorting nexin-3
203	4.938271642	IPI00815770,2	SNX3 Isoform 1 of Sorting nexin-3
204	8.333333582	IPI00795720,1	CD63 13 kDa protein
204	8.333333582	IPI00791992,1	CD63 14 kDa protein
204	7.692307979	IPI00791162,1	CD63 14 kDa protein
204	7.042253762	IPI00795188,1	CD63 15 kDa protein
204	4.651162773	IPI00384548,2	CD63 Lysosome-associated membrane protein-3 variant
204	4.237288237	IPI00749070,1	CD63 CD63 antigen isoform B
204	4.201680794	IPI00215998,5	CD63 CD63 antigen
205	7.361963391	IPI00792746,1	GRB2 19 kDa protein
205	6.818182021	IPI00218070,1	GRB2 Isoform GRB3-3 of Growth factor receptor-bound protein 2
205	5.529953912	IPI00021327,3	GRB2 Isoform 1 of Growth factor receptor-bound protein 2
206	5.434782431	IPI00791637,1	UNQ9391 Similar to Tryptophan/serine protease
206	4.26136367	IPI00394814,4	UNQ9391 Probable serine protease UNQ9391/PRO34284
207	3.669724613	IPI00216393,1	CLTA Isoform Non-brain of Clathrin light chain A

207	3.389830515	IPI00790571,2	CLTA Isoform 3 of Clathrin light chain A
207	3.225806355	IPI00014587,1	CLTA Isoform Brain of Clathrin light chain A
208	2.476780117	IPI00789155,1	CALU Calumenin, isoform CRA_c
209	1.031991746	IPI00293033,5	NID2 NID2 protein
209	0.847457629	IPI00745450,1	NID2 Putative uncharacterized protein DKFZp686D12108 (Fragment)
209	0.727272732	IPI00028908,3	NID2 Nidogen-2
210	5.999999866	IPI00644896,1	EXOSC2 Exosome component 2
210	4.562737793	IPI00647528,1	EXOSC2 Exosome component 2
210	4.444444552	IPI00645362,1	EXOSC2 Exosome component 2
210	4.210526496	IPI00646511,1	EXOSC2 Exosome component 2
210	4.095563293	IPI00015905,1	EXOSC2 Exosome complex exonuclease RRP4
211	9.271523356	IPI00644994,1	FBP1 Fructose-1,6-bisphosphatase 1
211	4.142011702	IPI00073772,5	FBP1 Fructose-1,6-bisphosphatase 1
212	2.708333358	IPI00021794,8	CTSA Lysosomal protective protein
212	2.610441856	IPI00640525,2	CTSA cathepsin A isoform a precursor
213	3.092783503	IPI00386702,1	NUP54 Isoform 2 of Nucleoporin p54
213	1.77165363	IPI00172580,4	NUP54 Nucleoporin 54kDa variant (Fragment)
214	2.096435986	IPI00337494,7	SLC25A24 Isoform 1 of Calcium-binding mitochondrial carrier protein SCaMC-1
215	2.092050202	IPI00298971,1	VTN Vitronectin
216	6.557376683	IPI00297579,4	CBX3;LOC653972 Chromobox protein homolog 3
217	0.99290777	IPI00297492,2	STT3A Dolichyl-diphosphooligosaccharide--protein glycosyltransferase subunit STT3A
218	2.578796633	IPI00295777,6	GPD1 Glycerol-3-phosphate dehydrogenase [NAD+], cytoplasmic
219	4.729729891	IPI00294158,1	BLVRA Biliverdin reductase A
220	1.264044922	IPI00103356,2	ITGB2 Integrin beta
220	1.170351077	IPI00291792,2	ITGB2 Integrin beta-2
221	1.632047445	IPI00218916,3	ALOX5 Arachidonate 5-lipoxygenase
222	2.813299187	IPI00165579,6	CNDP2 CNDP dipeptidase 2 (Metallopeptidase M20 family), isoform CRA_b
222	2.315789461	IPI00177728,3	CNDP2 Cytosolic non-specific dipeptidase
223	3.205128387	IPI00171390,2	PRPF38A Isoform 1 of Pre-mRNA-splicing factor 38A
224	1.982378773	IPI00093057,6	CPOX Coproporphyrinogen III oxidase, mitochondrial
225	11.20000035	IPI00032827,1	SF3B14 Pre-mRNA branch site protein p14
226	3.365384787	IPI00031131,4	C20orf3 Adipocyte plasma membrane-associated protein
227	2.037036978	IPI00027851,2	HEXA cDNA FLJ53927, highly similar to Beta-hexosaminidase alpha chain

228	4.137931019	IPI00024993,4	ECHS1 Enoyl-CoA hydratase, mitochondrial
229	7.228915393	IPI00012011,6	CFL1 Cofilin-1
230	3.389830515	IPI00009253,2	NAPA Alpha-soluble NSF attachment protein
231	2.431118302	IPI00007682,2	ATP6V1A V-type proton ATPase catalytic subunit A
232	6.511627883	IPI00006865,3	SEC22B Vesicle-trafficking protein SEC22b
233	11.81818172	IPI00005511,1	PHF5A PHD finger-like domain-containing protein 5A
234	6.596305966	IPI00853455,1	CTSD Protein
234	6.067961082	IPI00011229,1	CTSD Cathepsin D
234	5.351170525	IPI00852597,1	CTSD 33 kDa protein
235	7.086614519	IPI00878638,1	- 14 kDa protein
235	4.16883789	IPI00736284,1	SDK2 Isoform 2 of Protein sidekick-2
235	3.719200194	IPI00384376,3	SDK2 Isoform 3 of Protein sidekick-2
235	3.686635941	IPI00292043,6	SDK2 Isoform 1 of Protein sidekick-2
235	3.613369539	IPI00478949,4	SDK2 Isoform 4 of Protein sidekick-2
235	1.960784383	IPI00902868,1	SDK1 Isoform 3 of Protein sidekick-1
235	1.943063736	IPI00784490,1	SDK1 Isoform 1 of Protein sidekick-1
235	1.93868354	IPI00383951,6	SDK1 Putative uncharacterized protein SDK1
236	12.5	IPI00607716,1	PSMD5 8 kDa protein
236	1.952277683	IPI00909766,1	- cDNA FLJ52877, highly similar to 26S proteasome non-ATPase regulatory subunit 5
236	1.785714366	IPI00002134,4	PSMD5 26S proteasome non-ATPase regulatory subunit 5
237	7.416267693	IPI00028091,3	ACTR3 Actin-related protein 3
238	2.991452999	IPI00872773,1	ERO1L Putative uncharacterized protein ERO1L
238	2.991452999	IPI00386755,2	ERO1L ERO1-like protein alpha
239	2.393340319	IPI00328550,3	THBS4 Thrombospondin-4
240	1.482213475	IPI00017283,2	IARS2 Isoleucyl-tRNA synthetase, mitochondrial
241	15.65217376	IPI00514997,1	NANS N-acetylneuraminic acid synthase
241	12.85714358	IPI00514469,1	NANS N-acetylneuraminic acid synthase
241	5.013927445	IPI00147874,1	NANS Sialic acid synthase
242	6.687898189	IPI00740620,2	WDR38 WD repeat-containing protein 38
243	17.24137962	IPI00215780,5	RPS19 40S ribosomal protein S19
244	8.561643958	IPI00642697,4	NOP56 Nucleolar protein 5A
244	4.208754376	IPI00411937,4	NOP56 Nucleolar protein 5A
