# Supplementary figures and images for: A novel method for sample preparation of fresh lung cancer tissue for proteomics analysis by tumor cell enrichment and removal of blood contaminants
Source: Proteome Sci. 2010 Feb 26;8:9. doi: 10.1186/1477-5956-8-9 (PMC2847553; doi:10.1186/1477-5956-8-9)

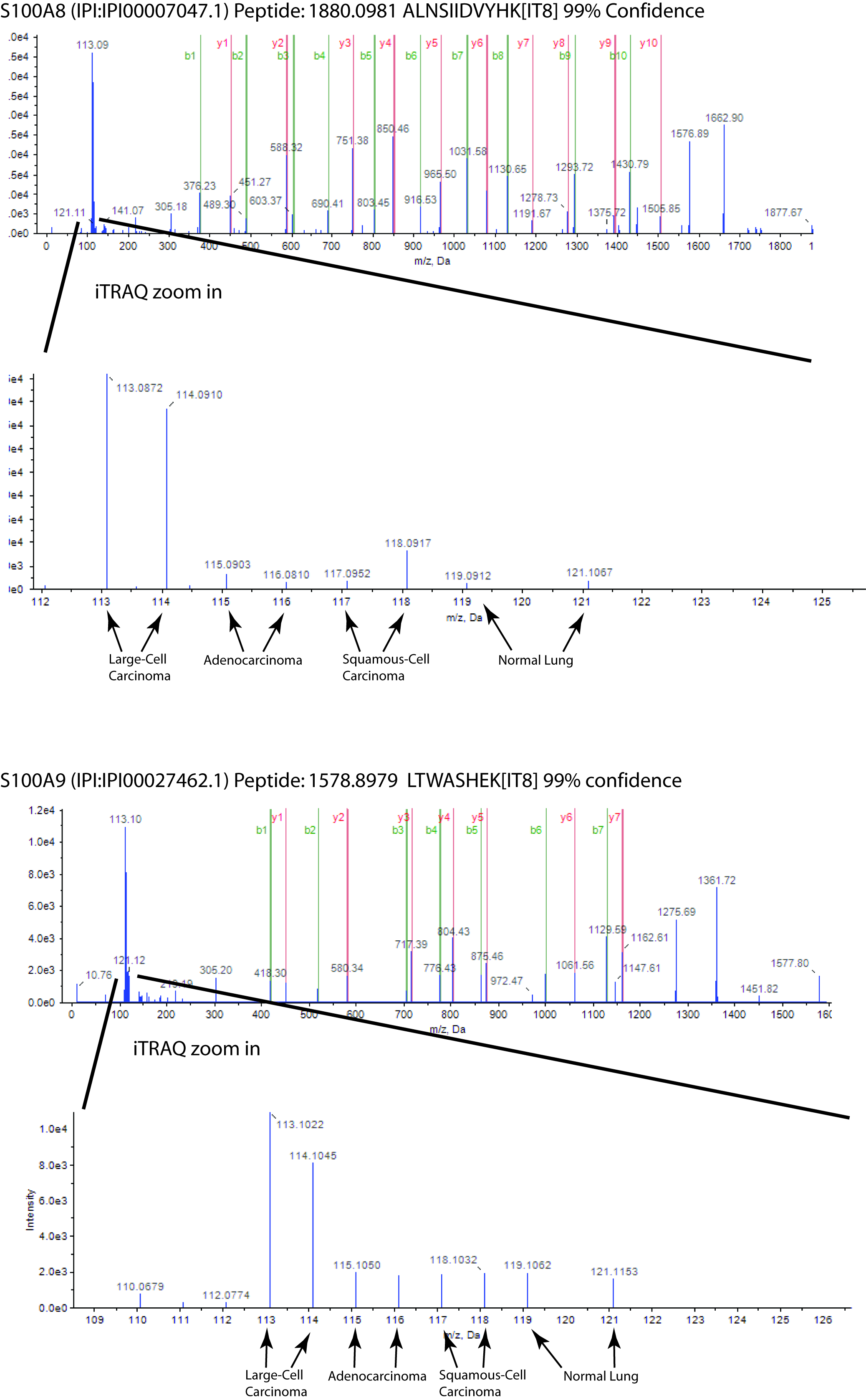

Supplement: Additional file 3 — Representative iTRAQ spectra. Representative iTRAQ spectra of one of the peptides from the proteins S100A8 and S100A9 identified by MS/MS analysis of lysates from the ETS preparation showing a higher relative abundance of these proteins in the samples of large-cell tumors compared with the other histological types and with the normal lung samples. [file 1477-5956-8-9-S3.TIFF]
